# Socioeconomic and ethnic disparities in preterm births in an English maternity setting: a population-based study of 1.3 million births

**DOI:** 10.1186/s12916-024-03493-x

**Published:** 2024-09-20

**Authors:** G. Kayode, A. Howell, C. Burden, R. Margelyte, V. Cheng, M. Viner, J. Sandall, J. Carter, L. Brigante, C. Winter, F. Carroll, B. Thilaganathan, D. Anumba, A. Judge, E. Lenguerrand, Tommy’s National Centre for Maternity Improvemen﻿t

**Affiliations:** 1 Translational Health Science, Bristol Medical School, University of Bristol, Southmead Hospital, Bristol, BS105NB UK; 2 Department of Women and Children’s Health, School of Life Course & Population Sciences, King’s College London, London, UK; 3 Royal College of Midwives, London, UK; 4 Department of Women’s Health, The PROMPT Maternity Foundation, Southmead Hospital, Bristol, UK; 5 Royal College of Obstetricians and Gynaecologists, London, UK; 6 Tommy’s National Centre for Maternity Improvement, Royal College of Obstetricians and Gynaecologists, 10-18 Union Street, London, SE1 1SZ UK; 7 Academic Unit of Reproductive and Developmental Medicine, University of Sheffield, Sheffield, UK

**Keywords:** Disparity, Preterm birth, Ethnicity, Health inequalities

## Abstract

**Background:**

Preterm birth is a major cause of infant mortality and morbidity and accounts for 7–8% of births in the UK. It is more common in women from socially deprived areas and from minority ethnic groups, but the reasons for this disparity are poorly understood. To inform interventions to improve child survival and their quality of life, this study examined the socioeconomic and ethnic inequalities in preterm births (< 37 weeks of gestation at birth) within Health Trusts in England.

**Methods:**

This study investigated socioeconomic and ethnic inequalities in preterm birth rates across the National Health Service (NHS) in England. The NHS in England can be split into different units known as Trusts. We visualised between-Trust differences in preterm birth rates. Health Trusts were classified into five groups based on their standard deviation (SD) variation from the average national preterm birth rate. We used modified Poisson regression to compute risk ratios (RR) and 95% confidence intervals (95% CI) with generalised estimating equations.

**Results:**

The preterm birth rate ranged from 6.8/100 births for women living in the least deprived areas to 8.8/100 births for those living in the most deprived areas. Similarly, the preterm birth rate ranged from 7.8/100 births for white women, up to 8.6/100 births for black women. Some Health Trusts had lower than average preterm birth rates in white women whilst concurrently having higher than average preterm birth rates in black and Asian women. The risk of preterm birth was higher for women living in the most deprived areas and ethnicity (Asian).

**Conclusions:**

There was evidence of variation in rates of preterm birth by ethnic group, with some Trusts reporting below average rates in white ethnic groups whilst concurrently reporting well above average rates for women from Asian or black ethnic groups. The risk of preterm birth varied substantially at the intersectionality of maternal ethnicity and the level of socioeconomic deprivation of their residency. In the absence of other explanations, these findings suggest that even within the same Health Trust, maternity care may vary depending on the women’s ethnicity and/or whether she lives in an area of high socioeconomic deprivation. Thus, social factors are likely key determinants of inequality in preterm birth rather than provision of maternity care alone.

**Supplementary Information:**

The online version contains supplementary material available at 10.1186/s12916-024-03493-x.

## Background

The incidence of preterm birth continues to increase globally, including in most European countries [1, 2]. Besides being the highest contributor to neonatal deaths [1, 3], preterm babies are more vulnerable to multiple medical conditions, including respiratory, gastrointestinal, cardiovascular, haematological, neurological and metabolic disorders [4]. Therefore, reducing the occurrence of preterm birth is an urgent global health priority, given its impact on childhood mortality and life-long morbidity [3, 5]. Though Europe only accounts for 4.7% of the global burden of preterm birth [1], 1 in every 14 births is a preterm birth, and over 50,000 cases occur annually in the United Kingdom (UK) [6–8].

Due to the persistent burden of preterm birth, the UK Secretary of State for Health pledged to reduce the preterm birth rate from 8 to 6% by 2025 within England [9]. In response, the Saving Babies’ Lives Care Bundle [10] was revised to include recommendations for Integrated Care Boards and national guidelines on providing best practice pathways that predict, prevent and prepare women at high risk of preterm birth [11]. Consequently, most studies in high-income countries (HICs), including the UK, have worked extensively on identifying the risk factors for preterm birth [4, 12–14], predicting preterm births [15, 16] and improving the survival of preterm babies [17, 18]. However, the persistent increase in preterm birth rates, despite existing preventive measures in women deemed to be at high risk, such as cervical cerclage, prophylactic progesterone, pessaries, aspirin intake and antibiotic administration, is concerning [19–22]. An approach to addressing this ongoing challenge is to focus on understanding and redressing the care variations and social inequities that may account for much of the preterm birth burden in the UK. As a high-income country, the higher incidence of preterm births has been linked to increased rates of late preterm births due to obstetric interventions such as caesarean sections and inductions of labour [23], with higher numbers of caesarean sections occurring in more affluent populations [24]. Strategies to avoid the use of non-medically indicated inductions and caesarean sections would help to reduce preterm birth rates.

As observed between countries [1, 25–27], the Office for National Statistics data indicates the possibility of regional variation in preterm births in the UK [8]. Significant socioeconomic disparities are reported within England, which are known to disproportionately affect women [28]. There is however a paucity of evidence regarding how the pattern and distribution of these disparities affect preterm birth rates. This study aimed to describe the variation in preterm birth rates by ethnic group and social deprivation within individual NHS Trusts and identify the risk factors of preterm birth, to inform targeted strategies to narrow these inequalities [29]. It also aimed to establish if high preterm rates reflected in some NHS Health Trusts was due to the issues around in utero transfer of women to Trusts with greater neonatal care facilities.

## Methods

### Study design and participants

This observational study utilised maternity care records from mothers and babies born in England between April 1, 2015, and March 31, 2017. Care in NHS England is delivered by organisational units called NHS Trusts. Each Trust serves a specific geographical catchment area employing uniform clinical guidelines. All 1,174,047 live births, of at least 24 weeks of gestation, in 130 NHS Trusts were eligible for inclusion.

### Data sources and linkage

We analysed the Maternity Information Systems (MIS) data, which collates routinely collected data from English NHS hospitals, by the National Maternity and Perinatal Audit (NMPA) (https://maternityaudit.org.uk/pages/home.) following approval from the Healthcare Quality Improvement Partnership (DARS-NIC-430380-F7L4Z-v0.4 HQIP348). During the study period, 130 Health Trusts submitted specific maternity information to the NMPA registry in England. The MIS datasets cover about 97% of all total births in England, and the data are of high quality [30, 31].

In England, deprivation is measured in small geographical areas known as Lower Layer Super Output Area (LSOA) [32]. LSOAs are defined as geographical areas of a similar population size, with an average of 1500 residents that preserves participant residential information confidentiality. As a measure of socioeconomic deprivation, we used the Index of Multiple Deprivation (IMD) score, a publicly available measure of deprivation available for each LSOA produced by the Office of National Statistics [32, 33]. The LSOA information, in the MIS dataset, was used to link the Office for National Statistics (ONS) IMD information to each maternal residential area. This is a combination several postcode areas and therefore preserves participant residential information confidentiality.

### Variables of interest

Maternal ethnicity was reported as recorded by healthcare providers in the MIS dataset. IMD, an aggregated index of socioeconomic deprivation of the maternal residential area, was considered a proxy for maternal socioeconomic status [33].

### Outcomes

Preterm birth was defined as a baby born before completing 37 weeks of gestation. Preterm births before 34 and then before 28 weeks of gestation were also investigated and reported in the supplementary.

#### Covariates

We classified ethnic groups as Asian, black, mixed ethnicity, white and any other ethnic group. We categorised IMD into five groups (quintiles), with 1 being the most deprived socioeconomic area and 5 denoting the least deprived socioeconomic group. The seven domains of deprivation used to generate deprivation scores include income, employment, education, health, crime, barriers to housing and services, and living environment. Maternal characteristics of interest included maternal body mass index, maternal age, maternal smoking status at booking, maternal alcohol consumption at booking, maternal substance abuse at booking, maternal mental health problems at booking, maternal domestic abuse at booking, previous total number of births, the number of complications diagnosed at booking, previous caesarean section, previous stillbirth, previous preterm birth, previous low weight infant and previous stillbirth.

### Statistical analysis

First, disparities in preterm birth rates were calculated for Health Trusts. The mean rate of preterm birth and the standard deviation (SD) was determined across all Trusts included in the analysis. Using the national mean rate of preterm birth and corresponding SD, Trusts were classified into five categories based on their preterm birth rates. They were categorised as “well below average” if the rate of preterm was more than 2 SDs below the national mean preterm rate (mean preterm rate minus 2*SD) ([< − 2SD], shown in green within all figures), “below average” (− 2SD to − 1SD below the national average, dark blue), “average” (− 1SD to + 1SD around the national average, sky blue), “above average” (+ 1SD to + 2SD above the national average, orange) and “well above average” (> + 2SD above the national average, red). Average preterm birth rates estimated by maternal ethnicity and IMD were compared to the national average.

Second, we calculated the absolute risk of preterm birth. We performed a one-sample *t*-test to compare the average absolute risk of preterm birth for each group compared to the national average.

These analyses were repeated in sensitivity analyses where we redefined the outcome of interest as preterm birth defined by a baby born before completing 34 weeks of gestation and 28 weeks of gestation.

Lastly, we used Zou’s modified Poisson regression to establish the effect of ethnicity and IMD on preterm birth [34]. We accounted for clustering in the data by Health Trust by applying the sandwich variance estimator for clustered data [35]. Variables were entered into the multivariable model if they had known clinical relevance [36, 37]. Missing data were imputed using multiple imputation by chained equations under the missing at random assumption [38]. We created 25 complete data sets, pooling results using Rubin’s rules [39]. Results were presented as risk ratios (RR) and 95% confidence intervals (95% CI). Association strength was interpreted as per [40].

All statistical analyses were performed in RStudio statistical software package version 4.0.2.

## Results

The maternal characteristics for the 1,174,047 live births and 91,056 preterm births (7.8 preterm births per 100 live births) captured during the study period are shown in Table 1 and Figure S1. The highest proportions of preterm births occurred in nulliparous women (42.8%, *n* = 35,024), aged 30 to 34 years (30%, *n* = 27,202) and in those with a BMI between 18.5 and 25 kg/cm^2^ (45.9%, *n* = 31,625). Similarly, the highest percentage of preterm births occurred in white women (70%, *n* = 63,636) and in those living in the most deprived areas (30%, *n* = 25,888). However, the highest rates of preterm birth (9.0/100 births and 8.8/100 births) were observed at the extremes of maternal age (< 20 years and ≥ 35 years), respectively, in women with five or more births (12.7/100 births) and those with a BMI < 18.5 kg/m^2^ (9.9/100 births). The preterm birth rate in Health Trusts with well above average rates (> + 2SDs) was 10.5/100 births.

**Table 1:** Sample description between April 2015 and March 2017

**Characteristics**	**All participants (*n* = 1,203,749)**	**Birth outcome (*n* = 1,174,047)**		
		**Term (*n* = 1,082,991, 92%)**	**Preterm**^**a**^** (*n* = 91,056, 8%)**	**Preterm birth rate (per 100 live births = 7.8)**
**Maternal age (years)**				
** < 20**	38,092	33,719 (91.0%)	3323 (9.0%)	9.0
** 20–24**	177,900	159,928 (92.2%)	13,520 (7.8%)	7.8
** 25–29**	337,655	305,457 (92.8%)	23,799 (7.2%)	7.2
** 30–34**	378,184	341,617 (92.6%)	27,202 (7.4%)	7.4
** ≥ 35**	265,858	236,665 (91.2%)	22,831 (8.8%)	8.8
** Missing data**^**b**^	6060 (0.5%^b^)	5602 (93.6%)	381 (6.4%)	6.4
**Parity**				
** 0 (nulliparous)**	444,254	400,072 (92.0%)	35,024 (8.1%)	8.1
** 1**	385,066	351,728 (93.6%)	24,180 (6.4%)	6.4
** 2**	158,912	143,068 (92.3%)	11,961 (7.7%)	7.7
** 3**	61,905	54,364 (90.2%)	5880 (9.8%)	9.8
** 4**	23,766	20,553 (89.0%)	2537 (11.0%)	11.0
** ≥ 5 (grand multiparous)**	18,332	15,528 (87.3%)	2267 (12.7%)	12.7
** Missing data**^**b**^	111,514 (9.3%^b^)	97,678 (91.4%)	9207 (8.6%)	8.6
**Body mass index (kg/m**^**2**^**)**				
** < 18.5**	27,822	24,455 (90.1%)	2680 (9.9%)	9.9
** 18.5 to < 25**	457,833	414,259 (92.9%)	31,625 (7.1%)	7.1
** 25 to < 30**	264,094	238,081 (92.6%)	18,955 (7.4%)	7.4
** 30 to < 35**	122,288	109,518 (92.1%)	9396 (7.9%)	7.9
** ≥ 35**	75,954	67,639 (91.5%)	6304 (8.5%)	8.5
** Missing data**^**b**^	255,758 (21.2%^b^)	229,039 (91.2%)	22,096 (8.8%)	8.8
**Ethnicity**				
** Asian**	130,326	115,287 (92.1%)	9939 (7.9%)	7.9
** Black**	54,819	48,665 (91.4%)	4592 (8.6%)	8.6
** Mixed**	20,457	18,301 (92.2%)	1550 (7.8%)	7.8
** Others**	46,218	41,703 (93.1%)	3107 (6.9%)	6.9
** White**	839,761	757,468 (92.2%)	63,636 (7.8%)	7.8
** Missing data**^**b**^	112,168 (9.3%^b^)	101,567 (92.5%)	8232 (7.5%)	7.5
**Index of Multiple Deprivation**				
** 1 (most deprived)**	303,006	269,796 (91.2%)	25,888 (8.8%)	8.8
** 2**	255,351	228,901 (92.1%)	19,543 (7.9%)	7.9
** 3**	212,959	191,637 (92.6%)	15,268 (7.4%)	7.4
** 4**	188,683	171,461 (92.9%)	13,083 (7.1%)	7.1
** 5 (least deprived)**	169,833	155,391 (93.2%)	11,362 (6.8%)	6.8
** Missing data**^**b**^	73,917 (6.1%^b^)	65,805 (91.8%)	5912 (8.2%)	8.2
**Maternity unit**				
** Well below average (< − 2SD)**	70,283	53,967 (97.0%)	1659 (3.0%)	3.0
** Below average (− 2SD to − 1SD)**	45,379	30,474 (94.5%)	1782 (5.5%)	5.5
** Average (− 1SD to + 1SD)**	978,242	864,897 (92.3%)	72,407 (7.7%)	7.7
** Above average (+ 1SD to + 2SD)**	145,109	119,559 (90.0%)	13,271 (10.0%)	10.0
** Well above average (> + 2SD)**	20,719	14,094 (87.9%)	1937 (12.1%)	12.1
**Health Trust**				
** Well below average (< − 2SD)**	30,564	28,549 (95.2%)	1443 (4.8%)	4.8
** Below average (− 2SD to − 1SD)**	132,679	117,925 (93.8%)	7786 (6.2%)	6.2
** Average (− 1SD to + 1SD)**	918,550	805,103 (92.2%)	67,673 (7.8%)	7.8
** Above average (+ 1SD to + 2SD)**	116,166	90,702 (90.6%)	9398 (9.4%)	9.4
** Well above average (> + 2SD)**	51,317	40,712 (89.5%)	4756 (10.5%)	10.5

Well below average: NHS Trusts with preterm birth rates at more than 2 SD below (< − 2SD) from the national rate of preterm birth in England; below average: NHS Trusts with preterm birth rates at − 2SD to − 1SD from the national rate; average: NHS Trusts with preterm birth rates at − 1SD to + 1SD from the national rate; above average: NHS Trusts with preterm birth rates at + 1SD to + 2SD from the national rate; well above average: NHS Trusts with preterm birth rates at > + 2SD from the national rate

*kg/m*^*2*^, kilogramme per square metre; *SD*, standard deviation

^a^Preterm birth was defined as baby born before completing 37 weeks of gestation

^b^Percentage of overall sample with missing information for the considered characteristic. Occurrence of missing data were observed to be similar in both arms of the outcome and < 10% in most cases

### Maternal characteristics

**Fig. 1:**
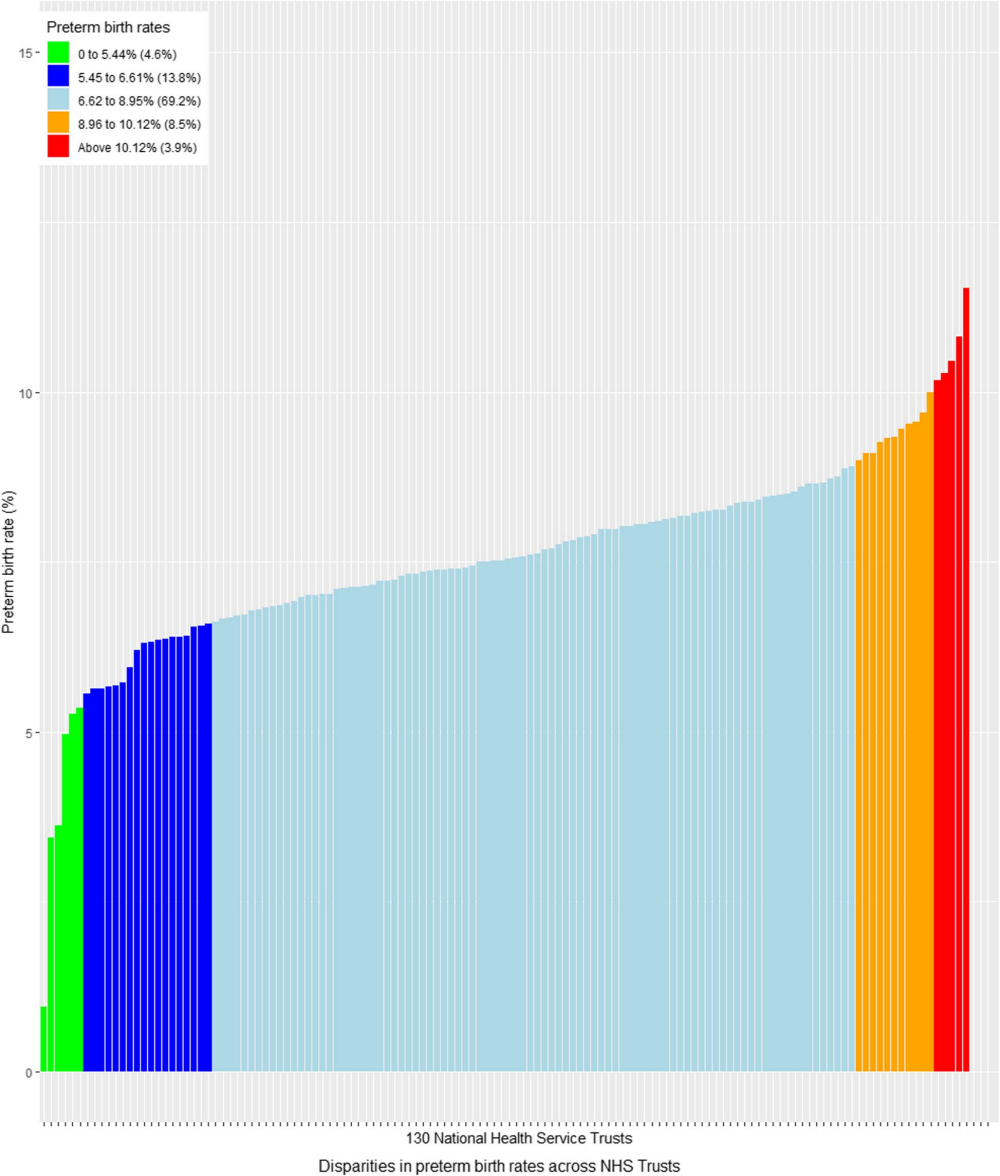
Preterm birth (< 37 weeks of gestation) rates across the 130 Health Trusts between April 2015 and March 2017

The variation in preterm birth rate across NHS Health Trusts in England is shown in Fig. 1. The proportion of Health Trusts categorised as well below the average rate for preterm births was 4.5% (green, *n* = 6), whereas the proportion of Health Trusts classified as well above the average rate was 3.8% (red, *n* = 5).

The maternal characteristics for the 1,174,047 live births and 25,604 preterm births at < 34 weeks of gestation (2.18 preterm births per 100 live births) and the 5346 preterm births at < 28 weeks of gestation (0.046 preterm births per 100 live births) are shown in Tables S1 and S2. The variation in preterm birth rate across Health Trusts in NHS England is consistent with the main analysis (Figures S2 and S3).

### Preterm birth rate by Health Trust of birth

The average preterm birth rates in Asian, black and white women in average Health Trusts (blue) were similar, 7.74/100 births, 7.60/100 births and 7.74/100 births, respectively (Table 2). Similarly, the average preterm birth rates across all ethnicities were comparable in below average Health Trusts (navy) and above average Health Trusts (orange). The corresponding figures within Health Trusts well above average (red) were 10.49/100 births, 11.62/100 births and 14.30/100 births, respectively. The average preterm birth rates for white, Asian and black women in the well below average (green) Health Trusts were 4.89/100 births, 3.46/100 births and 2.57/100 births, respectively (Table 2).

**Table 2:** Ethnic and socioeconomic disparities in absolute risk of preterm births across Health Trusts^a^

**Ethnicity**					
**Trusts**	**Asian**	**Black**	**Mixed race**	**Other races**	**White**
**Green**	3.46 (3.46; 3.47) (37, 3.2%)	2.57 (2.56; 2.58) (15, 1.3%)	3.25 (3.24; 3.26) (11, 0.9%)	3.97 (3.96; 3.97) (18, 1.5%)	4.89 (4.88; 4.90) (1081, 93%)
**Navy**	6.17 (6.17; 6.17) (981, 11.1%)	6.11 (6.11; 6.11) (400, 4.5%)	6.19 (6.19; 6.19) (138, 1.6%)	5.95 (5.95; 5.95) (225, 2.5%)	6.14 (6.14; 6.14) (7120, 80.3%)
**Blue**	7.60 (7.59; 7.60) (7138, 12.5%)	7.74 (7.73; 7.74) (3376, 5.9%)	7.86 (7.85; 7.86) (1139, 2.0%)	7.57 (7.57; 7.57) (2310, 4.0%)	7.74 (7.73; 7.74) (43,221, 75.6%)
**Orange**	9.49 (9.49; 9.60) (1203, 11.1%)	9.55 (9.55; 9.55) (678, 6.3%)	9.61 (9.61; 9.62) (177, 1.6%)	9.55 (9.55; 9.55) (428, 3.9%)	9.38 (9.38; 9.38) (8353, 77.0%)
**Red**	11.62 (11.61; 11.63) (580, 12.1%)	14.30 (14.28; 14.32) (123, 2.6%)	12.88 (12.86; 12.89) (85, 1.8%)	11.75 (11.74; 11.76) (126, 2.6%)	10.49 (10.48; 10.49) (3861, 80.1%)
**Socioeconomic Index of Multiple Deprivation (IMD)**					
**Trusts**	**IMD1 (most deprived)**	**IMD2**	**IMD3**	**IMD4**	**IMD5 (least deprived)**
**Green**	4.21 (4.20; 4.22) (331, 21.7%)	4.44 (4.43; 4.45) (339, 22.2%)	4.74 (4.73; 4.74) (331, 21.7%)	4.69 (4.69; 4.70) (281, 18.4%)	4.56 (4.56; 4.57) (200, 13.1%)
**Navy**	6.03 (6.03; 6.03) (1187, 12.6%)	6.13 (6.13; 6.13) (2003, 21.2%)	6.26 (6.26; 6.26) (1888, 20.0%)	6.04 (6.04; 6.04) (1970, 20.8%)	6.04 (6.04; 6.04) (1946, 20.6%)
**Blue**	8.13 (8.13; 8.12) (17,943, 28.5%)	7.82 (7.82; 7.82) (13,997, 22.2%)	7.78 (7.78; 7.78) (10,653, 16.9%)	7.56 (7.56; 7.56) (8857, 14.1%)	7.42 (7.42; 7.42) (7555, 12.0%)
**Orange**	9.39 (9.39; 9.39) (4013, 33.8%)	9.37 (9.37; 9.32) (2412, 20.3%)	9.36 (9.36; 9.36) (1745, 14.7%)	9.37 (9.37; 9.37) (1409, 11.9%)	9.39 (9.39; 9.40) (1104, 9.3%)
**Red**	12.63 (12.61; 12.65) (2414, 45.6%)	10.91 (10.91; 10.92) (792, 15.0%)	11.23 (11.22; 11.23) (651, 12.3%)	12.40 (12.38; 12.42) (566, 10.7%)	11.91 (11.89; 11.93) (557, 10.5%)

Preterm birth was defined as baby born before completing 37 weeks of gestation

All the estimated *P* values were < 0.00001

Green (well below average): NHS Trusts with preterm birth rate at < − 2 standard deviation (SD) from the national rate of preterm birth in England; navy (below average): NHS Trusts with preterm birth rate at − 2SD to − 1SD from the national rate; blue (average): NHS Trusts with preterm birth rates at − 1SD to + 1SD from the national rate; orange (above average): NHS Trusts with preterm birth rate at + 1SD to + 2SD from the national rate; red (well above average): Trusts with preterm birth rate at > + 2SD from the national rate

^a^Mean of NHS Trust preterm birth rates within a particular Trust preterm birth level, 95% confidence Interval, number and proportion of NHS Trusts

**Fig. 2:**
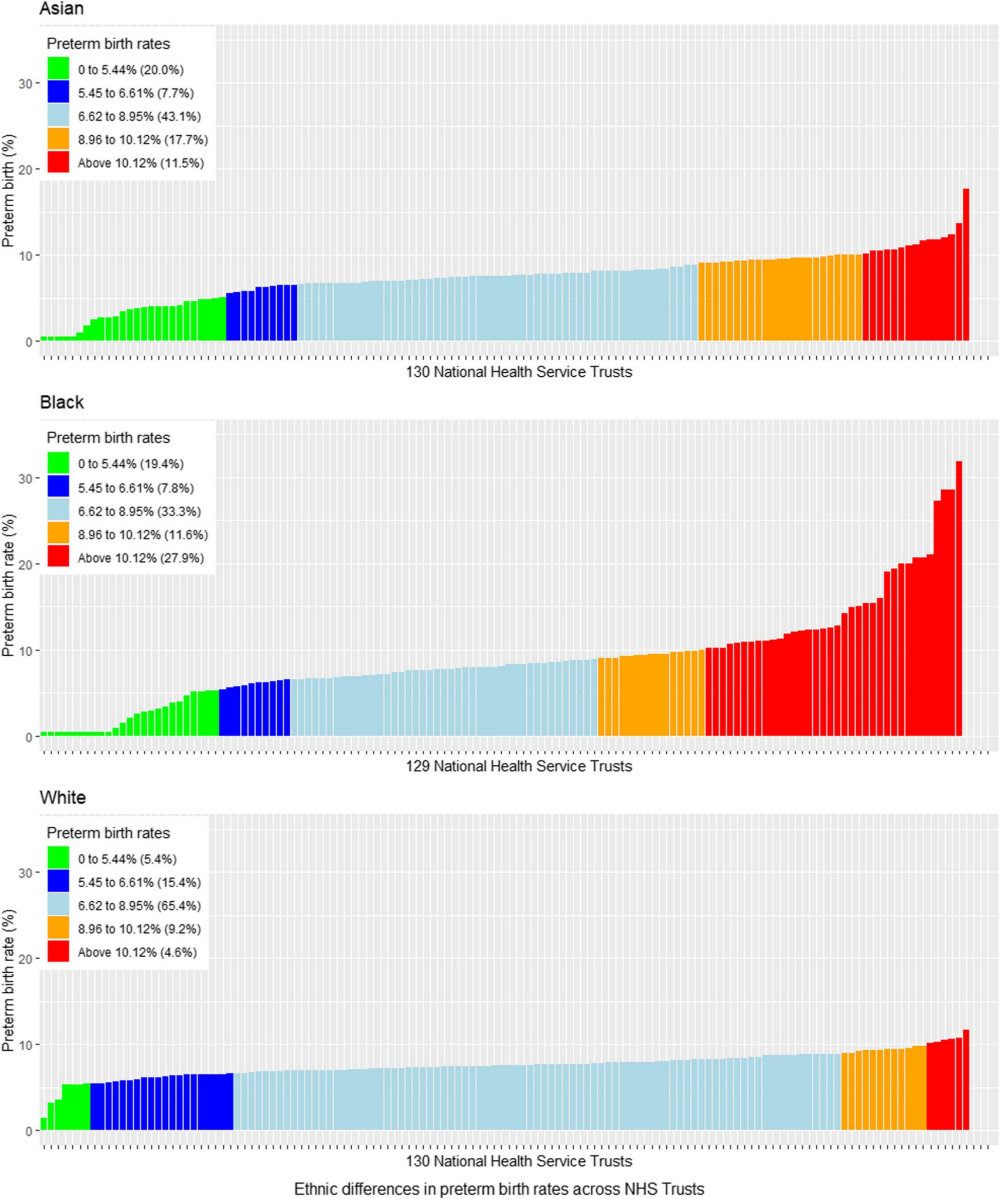
Preterm birth (< 37 weeks of gestation) rates by ethnicity across the 130 Health Trusts according to the national ethnic group preterm birth rates between April 2015 and March 2017

**Fig. 3:**
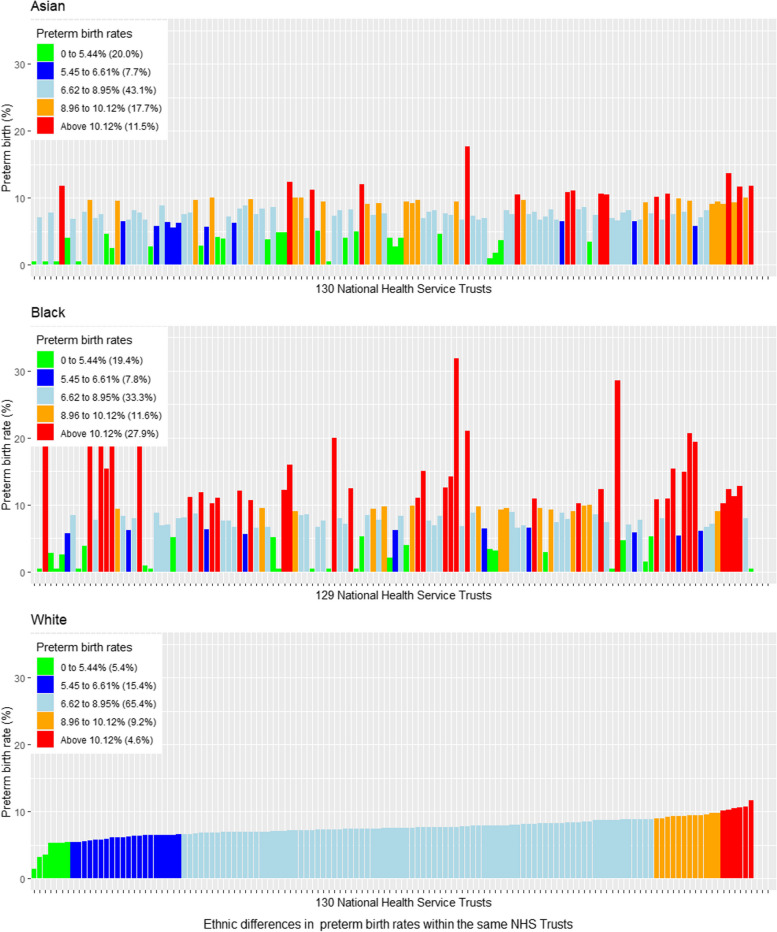
Preterm birth (< 37 weeks of gestation) rates by ethnicity across the 130 Health Trusts according to the overall national preterm birth rate between April 2015 and March 2017

**Fig. 4:**
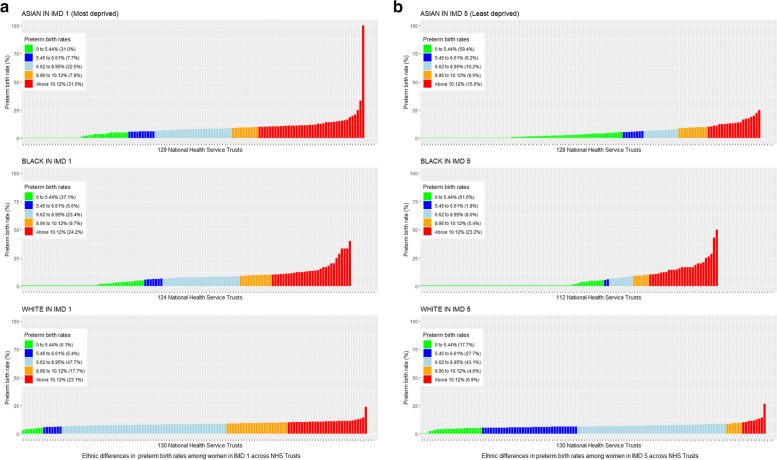
**a** Preterm birth (< 37 weeks of gestation) rates by ethnicity across the 130 Health Trusts according to the national ethnic group preterm birth rate within mums living in the most deprived areas (Index of Multiple Deprivation (IMD) 1) between April 2015 and March 2017. **b** Preterm birth (< 37 weeks of gestation) rates by ethnicity across the 130 Health Trusts according to the national ethnic group preterm birth rate within mums living in the least deprived areas (Index of Multiple Deprivation (IMD) 5) between April 2015 and March 2017

The proportion of Health Trusts with well above average (red) preterm birth rates in the general population was 3.2% (*n* = 5), with the equivalent proportion for white, Asian and black women being 4.6%, 11.5% and 27.9%, respectively (Fig. 2). When Health Trusts were ranked according to overall preterm birth rate, there were notable variations in rates of preterm birth rate within the same Trust for white, Asian and black women (Fig. 3). Some Health Trusts had below average preterm birth rates for white women whilst reporting average or well above average preterm rates for black and Asian women. The average rate of preterm birth was similar for all areas regardless of socioeconomic deprivation quintile when analysed by the average preterm birth rate of the trusts (Table 2), ranging from 7.42/100 births for women in the least deprived areas to 8.13/100 births for women living in the most deprived areas. For women living in the least deprived areas, the proportion of preterm births that occurred in Health Trusts with well above average preterm birth rates was 9.2%, whilst the corresponding figure was 2.3% for women living in the average deprived areas and 17.7% for those in the most deprived areas, respectively (Fig. 4a and b).

Ethnic inequalities within Health Trusts for women living in areas with the same level of socioeconomic deprivation were consistent with the main analysis when < 34 and < 28 weeks of gestation were considered to define preterm birth (Figures S4a, S4b, S5a and S5b). When Health Trusts were ranked according to overall preterm birth rate, there were notable variations in rates of preterm birth rate within the same Trust for white, Asian and black women (Figures S6 and S7).

### Preterm birth variation by ethnicity and/or IMD

The preterm birth rate ranged from a rate of 8.6/100 births in black women compared with a preterm birth rate of 6.9/100 births in women from other ethnic (non-black and non-Asian) groups (Table 1). Ethnic inequalities in preterm birth rates are displayed in Fig. 2. Similarly, the preterm birth rate ranged from a rate of 6.8/100 births for women living in the least deprived areas, up to a rate of 8.8/100 births for those living in the most deprived areas (Table 1). The average rate of preterm birth for women of different ethnic origins in various socioeconomic groups is shown in Table 2 and Fig. 4a and b. The preterm birth rates in Asian, black and white women from the most deprived areas were 8.5/100 births, 7.2/100 births and 7.7/100 births, respectively. The corresponding figures for white, Asian and black women from the least deprived areas were 6.3/100 births, 6.9/100 births and 6.6/100 births, respectively.

When preterm birth was redefined using 34 and 28 weeks of gestation rather than 37 weeks of gestation, the preterm birth variation by ethnicity (Figures S8 and S9 and Tables S3 and S4) and/or IMD (Figures S4a, S4b, S5a and S5b) were consistent with the main analysis. For example, black women had higher rates of preterm birth compared white women.

The associations between ethnicity and IMD with preterm birth are displayed in Table 3. Women of Asian ethnicity were more likely to experience preterm birth, compared to White women. No evidence of a difference could be identified for the other ethnic groups. Women residing in the most deprived areas were at an increased risk of preterm birth compared to those residing in the least deprived areas: As deprivation increased from IMD3 to IMD1, the risk of preterm increases in a dose–response manner.

**Table 3:** Adjusted relative risks of preterm birth by maternal ethnicity and IMD status^a^

	**Relative risk (95% CI)**^**b**^
**Ethnicity**	
White	Reference
Asian	1.05 (1.03–1.07)
Black	1.01 (0.98–1.04)
Mixed	0.96 (0.92–1.00)
Other	0.91 (0.88–0.95)
**Index of Multiple Deprivation**	
1—most deprived	1.26 (1.22–1.30)
2	1.15 (1.12–1.19)
3	1.09 (1.06–1.12)
4	1.05 (0.99–1.10)
5—least deprived	Reference

Preterm birth was defined as baby born before completing 37 weeks of gestation and classified as preterm vs term birth

^a^Modified Poisson regression adjusted for maternal body mass index, age, smoking status at booking, alcohol consumption at booking, substance abuse at booking, mental health problems at booking, domestic abuse at booking, previous total number of births, number of complications diagnosed at booking, previous caesarean section, previous stillbirth, previous preterm birth, previous low weight infant and previous stillbirth

^b^Analysis combining the estimations of the 25 imputed datasets using the Rubin’s rules; 95% CI=95% confidence Interval

## Discussion

### Main findings

This study investigated preterm birth across 130 NHS Health Trusts in England and found evidence of inequity in care provision within Health Trusts. Several Health Trusts report below average preterm birth rates for white women but concurrently report above average or well above average preterm birth rates for black women. In Health Trusts with preterm birth rates well above the average, black women had a greater average rate of preterm birth (14.30/100 births) compared with white women (10.49/100 births). Our study responded to one of the critical recommendations of previous studies that examined inter-country variation in preterm birth [1, 2, 25, 26]. The authors highlighted the importance of exploring within-country variation in preterm birth and identifying the underlying mechanisms driving it. Our observations are in accordance with prior studies in the UK showing that ethnic minorities and high levels of socioeconomic deprivation are directly related to rates of preterm birth [41, 42]. Socioeconomic disparities were independent risk factors for preterm birth in our adjusted regression, with women residing in IMD1 to IMD3 more likely to experience preterm birth compared to women residing in the least deprived areas (IMD5). Women of low socioeconomic status are more likely to face obstacles such as being disadvantaged and vulnerable [43]; therefore, poverty could be the leading factor preventing equal access to maternity care. To address inequalities in maternity care, enhancement in living standards for disadvantaged women is required to provide access to education and increase employment opportunities [44]. Addressing the complex association between preterm birth and socioeconomic deprivation will depend upon understanding these underlying patient-level factors influencing preterm birth [45]. Inequity in access to quality perinatal care due to mistrust of health services, language/communication difficulties, racial discrimination, poor nutrition, tobacco use, alcohol consumption and substance use could be central to these inequalities [46–50].

Given the substantial variation observed in preterm birth across Health Trusts, coupled with multiple studies that reported variation in perinatal care across trusts, it would be illogical to assume that the observed disparity could be attributed to patient-level factors alone [51, 52]. Both preterm birth clinics and neonatal units have well-developed tertiary level provision with a National Network to enable referral of women from secondary to tertiary level care [53]. There are well-established regional and national referral networks that coordinate the in utero transfer of pregnant mothers likely to deliver prematurely to the appropriate maternity facility with available neonatal cots and services and at the appropriate level of neonatal care for the anticipated severity of premature birth. It is to be expected that NHS Trusts with level 3 neonatal facilities will receive a greater number of in utero transfers of women at high risk of preterm birth. However, we established that 3 of the 5 Health Trusts with preterm birth rates greater than 2 SD above the national average did not have level 3 neonatal facilities. This highlights the importance for “targeted Health Trust” interventions where the high preterm birth rates could not be explained by the transfer of high-risk pregnancies in utero.

### Research and health policy implications

The observed ethnic and socioeconomic inequalities in preterm birth across Health Trusts have highlighted the importance of understanding the underlying patient-level and context-level (Health Trust) factors influencing preterm birth. Despite the highest observed preterm birth rates nested in particular maternal ethnic groups (Tables 1 and 2), some of these differences were no more evident in the analysis (Table 3) adjusting, among other factors, for deprivation (IMD). These “indirect” differences are likely to be due to factors nested in ethnic groups such as deprivation. The generic maternity care delivered in NHS Trusts could also play an important role and would require further tailoring to meet the clinical needs and underlying social issues nested among mothers of particular ethnic groups. This research describes the indirect clinical and public health inequalities that pregnant ethnic women are likely to experience in NHS maternity care services. Our findings identified populations where care should be closely monitored and reviewed to ensure everyone has access to the same interventions. This work demonstrates the need for local community engagement to reduce barriers for women with social risk factors, to address equity issues in maternity care in the UK.

Unlike most high-resource settings, in the UK, two tools have been developed [16, 54] to determine the likelihood of preterm birth in women with symptoms of threatened preterm labour so that care can be targeted appropriately (e.g. steroids and hospital admission/in utero transfer). In addition, these tools provide reassurance to women when the likelihood of preterm is low, therefore saving resources as well as reducing antenatal bed and neonatal cot blocking. The care offered reduces chance of neonatal mortality and morbidity but does not prevent preterm birth itself. Impact assessment on pregnancy outcome has been conducted [55] and QUiPP (QUantitative Innovation in Predicting Preterm Birth v.2 App), which accurately discriminates women who are at short-term risk of preterm birth, is recommended by the British Association of Perinatal Medicine.

### Strengths and limitations

The NMPA population-based data on birth outcomes in England used in this study is the most comprehensive official maternity service data set currently available. Whilst contextual inequalities in preterm births have been explored spatially/geographically previously in the UK [56, 57], this study went further, by exploring the differences in preterm birth rates across care providers. The study also considered markers of health inequality to uncover between-Trust and within-Trust differences. Furthermore, the estimation of the gestational age of the baby at booking, as employed in this data set, is a reliable assessment of gestational age (foetal crown-rump length) and is associated with an accuracy of ± 1 week, if conducted during the first trimester [58, 59].

However, there are also limitations as follows. This analysis includes preterm birth, which encompasses both spontaneous and medically induced occurrences (e.g. due to pre-eclampsia or foetal growth restriction). Differentiating between the two with the available data proves challenging. Interventions to tackle preterm birth differ depending on whether spontaneous or not, and it may be that these ethnic/social deprivation variations are different in these two groups. However, it is expected that the impact of this omission would have been largely limited using IMD metrics. This is a descriptive study which cannot establish a causal relationship. Also, based on available data, some markers of inequality such as migration status or education were not considered. Therefore, these factors could influence the inequalities observed. Additionally, we used IMD metrics to represent social deprivation, which is a broad measure and cannot provide information at the individual level. Furthermore, we were unable to sub-divide the broad ethnic groups and therefore could not examine internal variation within each ethnic group, thus potentially masking inequalities. It is possible that Trust “performance” is because of differing thresholds for curtailing pregnancy duration iatrogenically because of clinical indications. However, as we considered the presence of level 3 neonatal facilities within the 5 Health Trusts ranked as “red” for the general population, it is unlikely that high preterm birth rates were driven by a high rate of transfer to these Trusts. Despite the limitations of a retrospective design, our study highlights the data gaps that could be addressed through enhanced data capture and management strategies, at regional and national levels. Such enhancements will thus inform monitoring and evaluation of the impact of future interventions at health systems and organisational levels to reduce preterm birth rates and consequences.

## Conclusions

This study demonstrates that beyond ethnicity and/or socioeconomic deprivation, the location and services provided at the Health Trust of birth could play a major role in the inequity in health care delivered. This is a descriptive study which highlights inequalities that currently exist. Thus, disparities in preterm birth could be reduced by targeting populations that have higher than average rates of preterm birth as early as possible in the antenatal care pathway, as well as Health Trusts with demonstrable inequalities in care delivery.

## Supplementary Information

Additional file 1: Figure S1. Additional file 1 Data flow diagram. Figure S2. Preterm birth (<34 weeks of gestation) rates across the 130 Health Trusts between April 2015 and March 2017. Figure S3. Preterm birth (<28 weeks of gestation) rates across the 130 Health Trusts between April 2015 and March 2017. Figure S4a. Preterm birth (<34 weeks of gestation) rates by ethnicity across the 130 Health Trusts according to the national ethnic group preterm birth rate within mums living in the most deprived areas (Index of Multiple Deprivation (IMD) 1) between April 2015 and March 2017. Figure S4b. Preterm birth (<28 weeks of gestation) rates by ethnicity across the 130 Health Trusts according to the national ethnic group preterm birth rate within mums living in the least deprived areas (Index of Multiple Deprivation (IMD) 5) between April 2015 and March 2017. Figure S5a. Preterm birth (<34 weeks of gestation) rates by ethnicity across the 130 Health Trusts according to the national ethnic group preterm birth rate within mums living in the most deprived areas (Index of Multiple Deprivation (IMD) 1) between April 2015 and March 2017. Figure S5b. Preterm birth (<28 weeks of gestation) rates by ethnicity across the 130 Health Trusts according to the national ethnic group preterm birth rate within mums living in the least deprived areas (Index of Multiple Deprivation (IMD) 5) between April 2015 and March 2017. Figure S6. Preterm birth (<34 weeks of gestation) rates by ethnicity across the 130 Health Trusts according to the overall national preterm birth rate between April 2015 and March 2017. Figure S7. Preterm birth (<28 weeks of gestation) rates by ethnicity across the 130 Health Trusts according to the overall national preterm birth rate between April 2015 and March 2017. Figure S8. Preterm birth (<34 weeks of gestation) rates by ethnicity across the 130 Health Trusts according to the national ethnic group preterm birth rate between April 2015 and March 2017. Figure S9. Preterm birth (<28 weeks of gestation) rates by ethnicity across the 130 Health Trusts according to the national ethnic group preterm birth rate between April 2015 and March 2017. Table S1. Distribution of live births by maternal characteristics and preterm birth status at 34 weeks of gestation between April 2015 and March 2017. Table S2. Distribution of live births by maternal characteristics and preterm birth status at 28 weeks of gestation between April 2015 and March 2017. Table S3. Ethnic and socioeconomic disparities in absolute risk of preterm births (<34 weeks of gestation) across Health Trusts between April 2015 and March 2017. Table S4. Ethnic and socioeconomic disparities in absolute risk of preterm births (<28 weeks of gestation) across Health Trusts between April 2015 and March 2017.

## Data Availability

Restrictions apply to the availability of these data, which were used under licence for the current study, and so are not publicly available. The data that support this study will be available from the National Maternity and Perinatal Audit (NMPA) (https://maternityaudit.org.uk/pages/home)—following approval from Healthcare Quality Improvement Partnership.

## Abbreviations

BMI: Body mass index
DARS-NIC: Data Access Request Service
HICs: High-income countries
IMD: Index of Multiple Deprivation
LSOA: Lower Layer Super Output Area
MIS: Maternity Information Systems
NHS: National Health Service
NMPA: National Maternity and Perinatal Audit
ONS: Office for National Statistics
QUiPP App: QUantitative Innovation in Predicting Preterm Birth application
RR: Risk ratio
SD: Standard deviation
UK: United Kingdom
95% CI: 95% Confidence interval
