# Supplementary material for: Socioeconomic and ethnic disparities in preterm births in an English maternity setting: a population-based study of 1.3 million births
Source: BMC Med. 2024 Sep 20;22:371. doi: 10.1186/s12916-024-03493-x (PMC11414185; doi:10.1186/s12916-024-03493-x)
Supplement: Supplementary file 1 — Additional file 1: Figure S1. Additional file 1 Data flow diagram. Figure S2. Preterm birth (<34 weeks of gestation) rates across the 130 Health Trusts between April 2015 and March 2017. Figure S3. Preterm birth (<28 weeks of gestation) rates across the 130 Health Trusts between April 2015 and March 2017. Figure S4a. Preterm birth (<34 weeks of gestation) rates by ethnicity across the 130 Health Trusts according to the national ethnic group preterm birth rate within mums living in the most deprived areas (Index of Multiple Deprivation (IMD) 1) between April 2015 and March 2017. Figure S4b. Preterm birth (<28 weeks of gestation) rates by ethnicity across the 130 Health Trusts according to the national ethnic group preterm birth rate within mums living in the least deprived areas (Index of Multiple Deprivation (IMD) 5) between April 2015 and March 2017. Figure S5a. Preterm birth (<34 weeks of gestation) rates by ethnicity across the 130 Health Trusts according to the national ethnic group preterm birth rate within mums living in the most deprived areas (Index of Multiple Deprivation (IMD) 1) between April 2015 and March 2017. Figure S5b. Preterm birth (<28 weeks of gestation) rates by ethnicity across the 130 Health Trusts according to the national ethnic group preterm birth rate within mums living in the least deprived areas (Index of Multiple Deprivation (IMD) 5) between April 2015 and March 2017. Figure S6. Preterm birth (<34 weeks of gestation) rates by ethnicity across the 130 Health Trusts according to the overall national preterm birth rate between April 2015 and March 2017. Figure S7. Preterm birth (<28 weeks of gestation) rates by ethnicity across the 130 Health Trusts according to the overall national preterm birth rate between April 2015 and March 2017. Figure S8. Preterm birth (<34 weeks of gestation) rates by ethnicity across the 130 Health Trusts according to the national ethnic group preterm birth rate between April 2015 and March 2017. Figure S9. Prete [file 12916_2024_3493_MOESM1_ESM.zip › Additional file 1 newR3.docx]

Table S1 – Distribution of livebirths by maternal characteristics and preterm birth status at 34 weeks of gestation between April 2015 and March 2017

| **Characteristics** | | **Birth outcomes**  **(n = 1,174,047)** | |  |
| --- | --- | --- | --- | --- |
|  | | **Term Preterm(<34 weeks gestation)** | | **Preterm birth rate** |
|  | | **(n =** 1,148,443, 98%**)** | **(n =** 25,604, 2.2%**)** | **(Per 100 livebirths)** |
| **Maternal Age (years)**  <20  20 – 24  25 – 29  30 – 34  ≥35  Missing data* | | 36,017 (97.2%)  169,661 (97.8%)  322,704 (98.0%)  361,184 (97.9%)  252,988 (97.5%)  5,889 (98.4%) | 1,025 (2.8%)  3,787 (2.2%)  6,552 (2.0%)  7,635 (2.1%)  6,508 (2.5%)  97 (1.6%) | **2.8**  **2.2**  **2.0**  **2.1**  **2.5**  **1.6** |
| **Parity**  0 (Nulliparous)  1  2  3  ≥4  Missing data* | | 424,523 (97.6%)  369,747 (98.4%)  151,966 (98.0%)  58,636 (97.3%)  39,502 (96.6%)  104,069 (97.4%) | 10,573 (2.4%)  6,161 (1.6%)  3,063 (2.0%)  1,608 (2.7%)  1,383 (3.4%)  2,816 (2.6%) | **2.4**  **1.6**  **2.0**  **2.7**  **3.4**  **2.6** |
| **Body Mass Index (Kg/m^2^)**  <18.5  18.5 to <25  25 to <30  30 to <35  ≥35  Missing data* | | 26,412 (97.3%)  437,584 (98.1%)  251,864 (98.0%)  116,376 (97.9%)  72,247 (97.7%)  243,960 (97.1%) | 723 (2.7%)  8,300 (1.9%)  5,172 (2.0%)  2,538 (2.1%)  1,696 (2.3%)  7,175 (2.9%) | **2.7**  **1.9**  **2.0**  **2.1**  **2.3**  **2.9** |
| **Ethnicity**  Asian  Black  Mixed  Others  White  Missing data* | | 122,541 (97.9%)  51,665 (97.0%)  19,390 (97.7%)  43,937 (98.1%)  803,846 (97.9%)  107,064 (97.5%) | 2,685 (2.1%)  1,592 (3.0%)  461 (2.3%)  873 (1.9%)  17,258 (2.1%)  2,735 (2.5%) | **2.1**  **3.0**  **2.3**  **1.9**  **2.1**  **2.5** |
| **Index multiple deprivation**  1 (Most deprived)  2  3  4  5 (Least deprived)  Missing data* | | 288,390 (97.5%)  242,826 (97.7%)  202,652 (97.9%)  181,054 (98.1%)  163,671 (98.2%)  69,850 (97.4%) | 7,924 (2.5%)  5,618 (2.3%)  4,243 (2.1%)  3,490 (1.9%)  3,082 (1.8%)  1,867 (2.6%) | **2.5**  **2.3**  **2.1**  **1.9**  **1.8**  **2.6** |
| **Maternity Unit**  Well Below Average (<-2SD)  Below Average (-2SD to -1SD)  Average (-1SD to +1SD)  Above Average (+1SD to +2SD)  Well Above Average (>+2SD) | | 26,331 (99.8%)  100,783 (99.0%)  914,023 (97.8%)  91,859 (96.8%)  15,447 (94.3%) | 51 (0.2%)  1,063 (1.0%)  20,486 (2.2%)  3,073 (3.2%)  931 (5.7%) | **0.2**  **1.0**  **2.2**  **3.2**  **5.7** |
| **Health Trust**  Well Below Average (<-2SD)  Below Average (-2SD to -1SD)  Average (-1SD to +1SD)  Above Average (+1SD to +2SD)  Well Above Average (>+2SD) | | 7,883 (99.6%)  100,969 (98.8%)  893,780 (97.9%)  134,969 (96.9%)  10,842 (94.3%) | 29 (0.4%)  1,267 (1.2%)  19,335 (2.1%)  4,313 (3.1%)  660 (5.7%) | **0.4**  **1.2**  **2.1**  **3.1**  **5.7** |
| Occurrence of missing data were observed to be similar in both arms of the outcome and <10% in most cases  SD represents Standard deviation  Well below average (Green): Trusts with preterm birth rates at <-2SD from the national rate of preterm birth in England; Below average (Dark blue): Trusts with preterm birth rates at -2SD to -1SD from the national rate; Average (Sky blue): Trusts with preterm birth rates at -1SD to +1SD from the national rate; Above average (Orange): Trusts with preterm birth rates at +1SD to +2SD from the national rate; Well above average (Red): Trusts with preterm birth rates at >+2SD from the national rate | | | | |

Table S2: Distribution of livebirths by maternal characteristics and preterm birth status at 28 weeks of gestation between April 2015 and March 2017

| **Characteristics** | | **Birth outcomes**  **(n = 1,174,047)** | | | | | | |  | | |
| --- | --- | --- | --- | --- | --- | --- | --- | --- | --- | --- | --- |
|  | | | | **Term Preterm(<28 weeks gestation)** | | | **Preterm birth rate** | | |  |  |
|  | | | | **(n =** 1,168,701, 99.5%**)** | **(n =** 5,286, 0.5%**)** | | **(Per 100 livebirths)** | | |  |  |
| **Maternal Age (years)**  <20  20 – 24  25 – 29  30 – 34  ≥35  Missing data* | | \| 36,821 (99.4%) \| \| --- \| \| 172,672 (99.6%) \| \| 327,872 (99.6%) \| \| 367,162 (99.6%) \| \| 258,212 (99.5%) \| \| 5,962 (99.6%) \| | | | \| 221 (0.6%) \| \| --- \| \| 776 (0.4%) \| \| 1,384 (0.4%) \| \| 1,657 (0.4%) \| \| 1,284 (0.5%) \| \| 24 (0.4%) \| | | | **0.6**  **0.4**  **0.4**  **0.4**  **0.5**  **0.4** | | |  |
| **Parity**  0 (Nulliparous)  1  2  3  ≥4  Missing data* | | \| 432,851 (99.5%) \| 2,245 (0.5%) \| \| --- \| --- \| \| 374,666 (99.7%) \| 1,242 (0.3%) \| \| 154,437 (99.6%) \| 592 (0.4%) \| \| 59,909 (99.4%) \| 335 (0.6%) \| \| 40,606 (99.3%) \| 279 (0.7%) \| \| 106,232 (99.6%) \| 653 (0.4%) \| | | | |  | | | **0.5**  **0.3**  **0.4**  **0.6**  **0.7**  **0.4** | | |
| **Body Mass Index (Kg/m^2^)**  <18.5  18.5 to <25  25 to <30  30 to <35  ≥35  Missing data* | | \| 27,028 (99.6%) \| 107 (0.4%) \| \| --- \| --- \| \| 444,375 (99.7%) \| 1,509 (0.3%) \| \| 256,004 (99.6%) \| 1,032 (0.4%) \| \| 118,423 (99.6%) \| 491 (0.4%) \| \| 73,535 (99.4%) \| 408 (0.6%) \| \| 249,336 (99.3%) \| 1,799 (0.7%) \| | | | |  | | | **0.4**  **0.3**  **0.4**  **0.4**  **0.6**  **0.7** | | |
| **Ethnicity**  Asian  Black  Mixed  Others  White  Missing data* | | \| 124,683 (99.6%) \| 543 (0.4%) \| \| --- \| --- \| \| 52,802 (99.1%) \| 455 (0.9%) \| \| 19,733 (99.4%) \| 118 (0.6%) \| \| 44,615 (99.6%) \| 195 (0.4%) \| \| 817,750 (99.6%) \| 3,354 (0.4%) \| \| 109,118 (99.4%) \| 681 (0.6%) \| | | | |  | | | **0.4**  **0.9**  **0.6**  **0.4**  **0.4**  **0.6** | | |
| **Index multiple deprivation**  1 (Most deprived)  2  3  4  5 (Least deprived)  Missing data* | | \| 294,104 (99.5%) \| 1,580 (0.5%) \| \| --- \| --- \| \| 247,302 (99.5%) \| 1,142 (0.5%) \| \| 206,053 (99.6%) \| 852 (0.4%) \| \| 183,846 (99.6%) \| 698 (0.4%) \| \| 166,132 (99.6%) \| 621 (0.4%) \| \| 71,264 (99.4%) \| 453 (0.6%) \| | | | |  | | | **0.5**  **0.5**  **0.4**  **0.4**  **0.4**  **0.6** | | |
| **Maternity Unit**  Well Below Average (<-2SD)  Below Average (-2SD to -1SD)  Average (-1SD to +1SD)  Above Average (+1SD to +2SD)  Well Above Average (>+2SD) | | \| 0 (0.0%) \| 0 (0.0%) \| \| --- \| --- \| \| 0 (0.0%) \| 0 (0.0%) \| \| 1,109,624 (99.6%) \| 4,388 (0.4%) \| \| 48,407 (98.9%) \| 530 (1.1%) \| \| 10,670 (96.1%) \| 428 (3.9%) \| | | | |  | | | **NA**  **NA**  **0.4**  **1.1**  **3.9** | | |
| **Health Trust**  Well Below Average (<-2SD)  Below Average (-2SD to -1SD)  Average (-1SD to +1SD)  Above Average (+1SD to +2SD)  Well Above Average (>+2SD) | | | \| 0 (0.0%) \| 0 (0.0%) \| \| --- \| --- \| \| 0 (0.0%) \| 0 (0.0%) \| \| 1,114,747 (99.6%) \| 4,447 (0.4%) \| \| 42,892 (98.9%) \| 459 (1.1%) \| \| 11,062 (96.2%) \| 440 (3.8%) \| | | |  | | | **NA**  **NA**  **0.4**  **1.1**  **3.8** | | |
| Occurrence of missing data were observed to be similar in both arms of the outcome and <10% in most cases  SD represents Standard deviation  Well below average (Green): Trusts with preterm birth rates at <-2SD from the national rate of preterm birth in England; Below average (Dark blue): Trusts with preterm birth rates at -2SD to -1SD from the national rate; Average (Sky blue): Trusts with preterm birth rates at -1SD to +1SD from the national rate; Above average (Orange): Trusts with preterm birth rates at +1SD to +2SD from the national rate; Well above average (Red): Trusts with preterm birth rates at >+2SD from the national rate | | | | | | | | | | | |

Table S3 - Ethnic and socioeconomic disparities in absolute risk of preterm births (<34 weeks gestation) across Health Trusts between April 2015 and March 2017

| **ETHNICITY** | | | | | |
| --- | --- | --- | --- | --- | --- |
| Trust | Asian | Black | Mixed-race | Other Races | White |
| Green | 0.12 (0.12-0.12) | 0.0088 (0.0085-0.0090) | 0.079 (0.078-0.08) | 0.17 (0.17-0.17) | 0.33 (0.33-0.33) |
| Navy | 1.12 (1.12-1.13) | 1.30 (1.30-1.30) | 1.19 (1.18-1.19) | 1.18 (1.18-1.18) | 1.27 (1.27-1.27) |
| Blue | 2.18 (2.18-2.18) | 2.37 (2.37-2.37) | 2.16 (2.16-2.16) | 2.07 (2.07-2.07) | 2.11 (2.11-2.11) |
| Orange | 3.31 (3.31-3.31) | 3.12 (3.12-3.12) | 3.25 (3.25-3.25) | 3.28 (2.28-3.28) | 3.08 (3.08-3.09) |
| Red | 5.22 (5.21-5.23) | 6.21 (6.19-6.22) | 5.99 (5.98-6.00) | 4.56 (4.55-4.57) | 4.74 (4.73-4.75) |
| **SOCIOECONOMIC INDEX OF MULTIPLE DEPRIVATION (IMD)** | | | | | |
| Trust | IMD1 | IMD2 | IMD3 | IMD4 | IMD5 |
| Green | 0.16 (0.16-0.16) | 0.15 (0.152-0.157) | 0.50 (0.50-0.51) | 0.32 (0.32-0.33) | 0.46 (0.46-0.46) |
| Navy | 1.27 (1.27-1.27) | 1.30 (1.295-1.296) | 1.26 (1.26-1.26) | 1.23 (1.23-1.24) | 1.22 (1.22-1.22) |
| Blue | 2.25 (2.25-2.25) | 2.21 (2.206-2.207) | 2.14 (2.14-2.14) | 2.01 (2.01-2.01) | 2.08 (2.08-2.08) |
| Orange | 3.14 (3.14-3.14) | 3.15 (3.150-3.152) | 3.25 (3.25-3.25) | 3.30 (3.30-3.30) | 3.31 (3.31-3.31) |
| Red | 6.13 (6.11-6.15) | 4.65 (4.638-4.657) | 4.45 (4.44-4.45) | 6.35 (6.35-6.36) | 6.56 (6.53-6.59) |

| All the estimated P-value were <0.00001  SD represents Standard deviation |
| --- |
| Well below average (Green): Trusts with preterm birth rates at <-2SD from the national rate of preterm birth in England; Below average (Dark blue): Trusts with preterm birth rates at -2SD to -1SD from the national rate; Average (Sky blue): Trusts with preterm birth rates at -1SD to +1SD from the national rate; Above average (Orange): Trusts with preterm birth rates at +1SD to +2SD from the national rate; Well above average (Red): Trusts with preterm birth rates at >+2SD from the national rate |

Table S4 - Ethnic and socioeconomic disparities in absolute risk of preterm births (<28 weeks gestation) across Health Trusts between April 2015 and March 2017

| **ETHNICITY** | | | | | |
| --- | --- | --- | --- | --- | --- |
| Maternity units | Asian | Black | Mixed-race | Other Races | White |
| Blue | 0.299 (0.299-0.300) | 0.289 (0.288-0.290) | 0.226 (0.226-0.227) | 0.234 (0.233-0.234) | 0.371 (0.371-0.372) |
| Orange | 1.146 (1.145-1.147) | 1.173 (1.172-1.174) | 1.135 (1.134-1.135) | 1.208 (1.207-1.209) | NA |
| Red | 2.338 (2.329-2.348) | 2.873 (2.866-2.881) | 2.833 (2.820-2.847) | 1.912 (1.909-1.916) | 3.70 (3.70-3.70) |
| **SOCIOECONOMIC INDEX OF MULTIPLE DEPRIVATION (IMD)** | | | | | |
| Maternity units | IMD1 | IMD2 | IMD3 | IMD4 | IMD5 |
| Blue | 0.398 (0.398-0.399) | 0.387 (0.387-0.388) | 0.372 (0.372-0.373) | 0.299 (0.299-0.300) | 0.306 (0.306-0.307) |
| Orange | 1.073 (1.072-1.073) | 1.072 (1.071- 1.072) | 1.097 (1.096-1.099) | 1.174 (1.173-1.174) | 1.149 (1.149-1.150) |
| Red | 4.184 (4.163-4.206) | 3.689 (3.687-3.692) | 3.049 (3.034-3.064) | 3.899 (3.894-3.903) | 4.316 (4.288-4.344) |

| All the estimated P-value were <0.00001  SD represents Standard deviation |
| --- |
| Well below average (Green): Trusts with preterm birth rates at <-2SD from the national rate of preterm birth in England; Below average (Dark blue): Trusts with preterm birth rates at -2SD to -1SD from the national rate; Average (Sky blue): Trusts with preterm birth rates at -1SD to +1SD from the national rate; Above average (Orange): Trusts with preterm birth rates at +1SD to +2SD from the national rate; Well above average (Red): Trusts with preterm birth rates at >+2SD from the national rate |

Figure S1: Data flow diagram

Figure S2: Preterm birth (<34 weeks of gestation) rates across the 130 Health Trusts between April 2015 and March 2017

Figure S3: Preterm birth (<28 weeks of gestation) rates across the 130 Health Trusts between April 2015 and March 2017

Figure S4a: Preterm birth (<34 weeks of gestation) rates by ethnicity across the 130 Health Trusts according to the national ethnic group preterm birth rate within mums living in the most deprived areas (Index Multiple Deprivation(IMD) 1) between April 2015 and March 2017

Figure S4b: Preterm birth (<28 weeks of gestation) rates by ethnicity across the 130 Health Trusts according to the national ethnic group preterm birth rate within mums living in the least deprived areas (Index Multiple Deprivation(IMD) 5) between April 2015 and March 2017

Figure S5a: Preterm birth (<34 weeks of gestation) rates by ethnicity across the 130 Health Trusts according to the national ethnic group preterm birth rate within mums living in the most deprived areas (Index Multiple Deprivation(IMD) 1) between April 2015 and March 2017

Figure S5b: Preterm birth (<28 weeks of gestation) rates by ethnicity across the 130 Health Trusts according to the national ethnic group preterm birth rate within mums living in the least deprived areas (Index Multiple Deprivation(IMD) 5) between April 2015 and March 2017

Figure S6: Preterm birth (<34 weeks of gestation) rates by ethnicity across the 130 Health Trusts according to the overall national preterm birth rate between April 2015 and March 2017

Figure S7: Preterm birth (<28 weeks of gestation) rates by ethnicity across the 130 Health Trusts according to the overall national preterm birth rate between April 2015 and March 2017

Figure S8: Preterm birth (<34 weeks of gestation) rates by ethnicity across the 130 Health Trusts according to the national ethnic group preterm birth rate between April 2015 and March 2017

Figure S9: Preterm birth (<28 weeks of gestation) rates by ethnicity across the 130 Health Trusts according to the national ethnic group preterm birth rate between April 2015 and March 2017
