# Supplementary figures and images for: Socioeconomic and ethnic disparities in preterm births in an English maternity setting: a population-based study of 1.3 million births
Source: BMC Med. 2024 Sep 20;22:371. doi: 10.1186/s12916-024-03493-x (PMC11414185; doi:10.1186/s12916-024-03493-x)

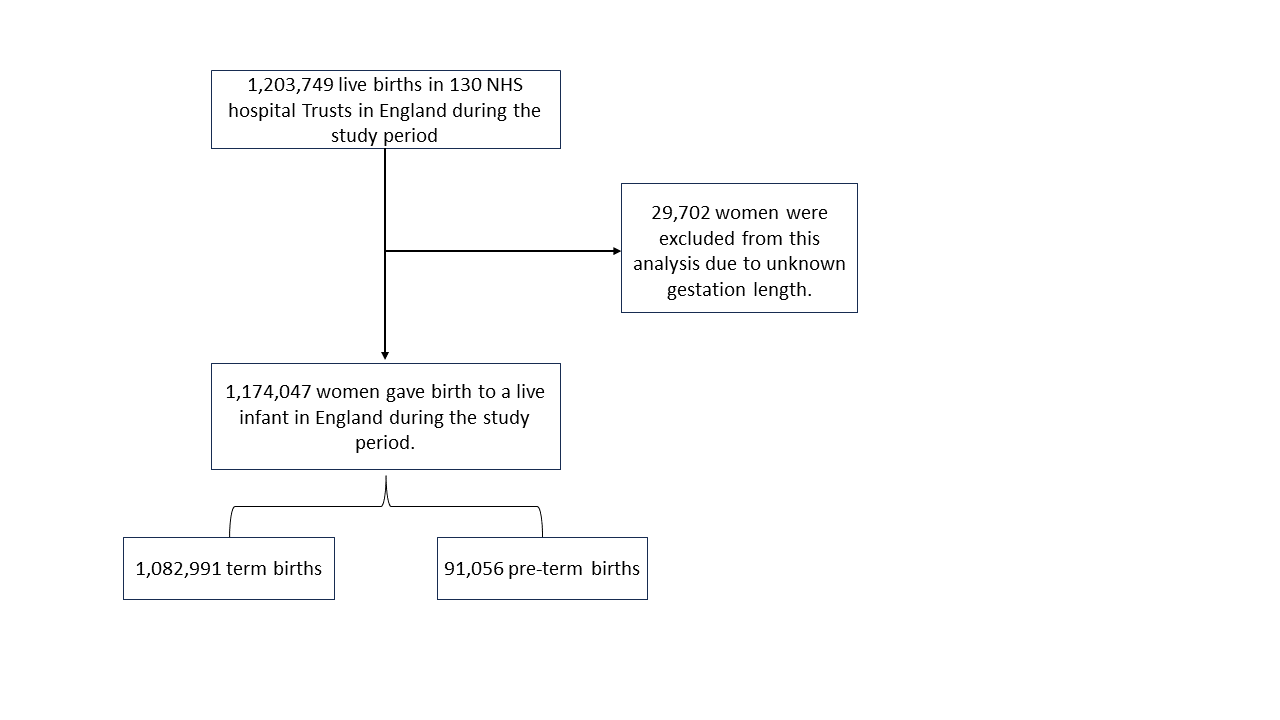

Supplement: Supplementary file 1 — Additional file 1: Figure S1. Additional file 1 Data flow diagram. Figure S2. Preterm birth (<34 weeks of gestation) rates across the 130 Health Trusts between April 2015 and March 2017. Figure S3. Preterm birth (<28 weeks of gestation) rates across the 130 Health Trusts between April 2015 and March 2017. Figure S4a. Preterm birth (<34 weeks of gestation) rates by ethnicity across the 130 Health Trusts according to the national ethnic group preterm birth rate within mums living in the most deprived areas (Index of Multiple Deprivation (IMD) 1) between April 2015 and March 2017. Figure S4b. Preterm birth (<28 weeks of gestation) rates by ethnicity across the 130 Health Trusts according to the national ethnic group preterm birth rate within mums living in the least deprived areas (Index of Multiple Deprivation (IMD) 5) between April 2015 and March 2017. Figure S5a. Preterm birth (<34 weeks of gestation) rates by ethnicity across the 130 Health Trusts according to the national ethnic group preterm birth rate within mums living in the most deprived areas (Index of Multiple Deprivation (IMD) 1) between April 2015 and March 2017. Figure S5b. Preterm birth (<28 weeks of gestation) rates by ethnicity across the 130 Health Trusts according to the national ethnic group preterm birth rate within mums living in the least deprived areas (Index of Multiple Deprivation (IMD) 5) between April 2015 and March 2017. Figure S6. Preterm birth (<34 weeks of gestation) rates by ethnicity across the 130 Health Trusts according to the overall national preterm birth rate between April 2015 and March 2017. Figure S7. Preterm birth (<28 weeks of gestation) rates by ethnicity across the 130 Health Trusts according to the overall national preterm birth rate between April 2015 and March 2017. Figure S8. Preterm birth (<34 weeks of gestation) rates by ethnicity across the 130 Health Trusts according to the national ethnic group preterm birth rate between April 2015 and March 2017. Figure S9. Prete [file 12916_2024_3493_MOESM1_ESM.zip › Additional file 1 Fig S1R3.png]

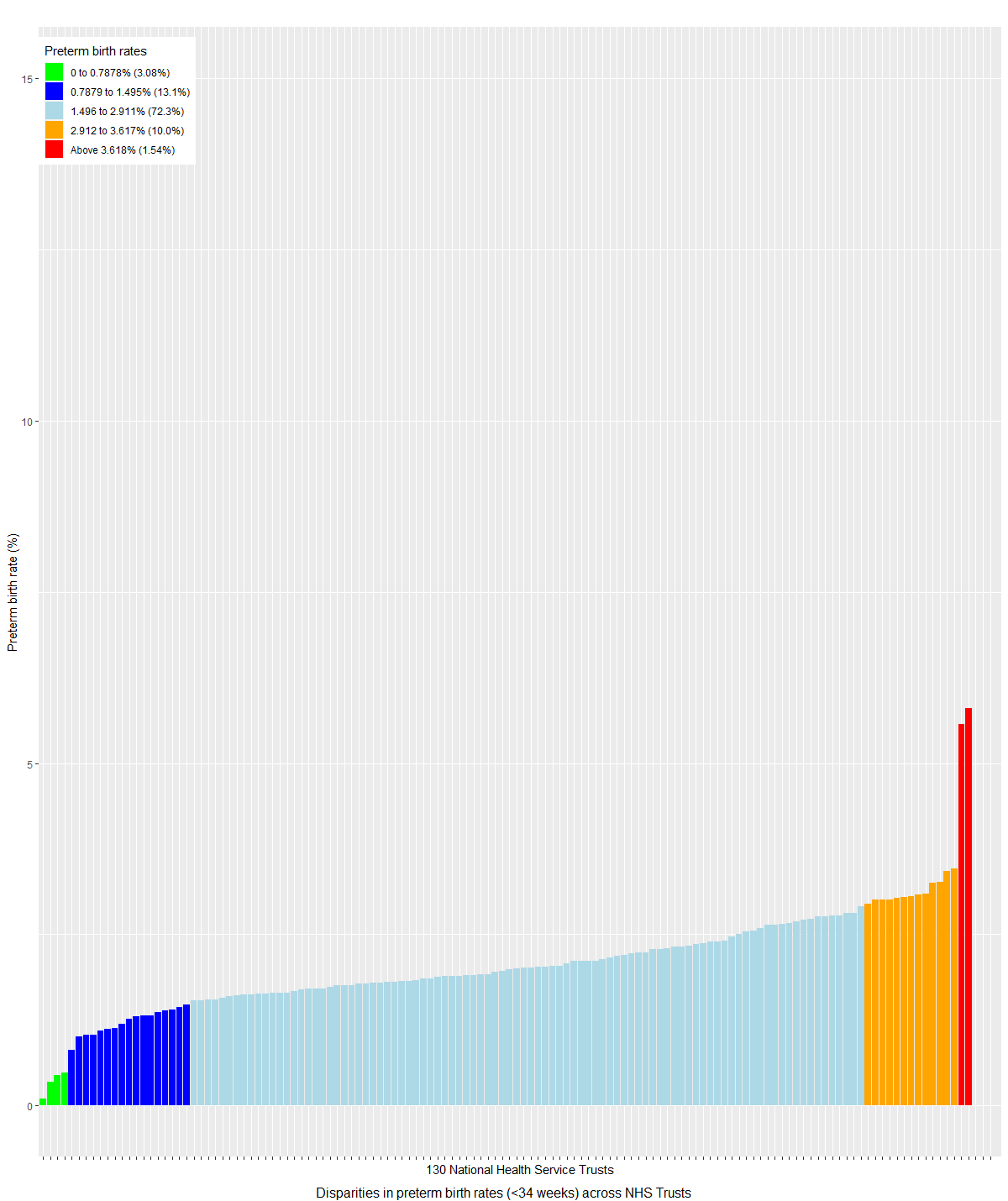

Supplement: Supplementary file 1 — Additional file 1: Figure S1. Additional file 1 Data flow diagram. Figure S2. Preterm birth (<34 weeks of gestation) rates across the 130 Health Trusts between April 2015 and March 2017. Figure S3. Preterm birth (<28 weeks of gestation) rates across the 130 Health Trusts between April 2015 and March 2017. Figure S4a. Preterm birth (<34 weeks of gestation) rates by ethnicity across the 130 Health Trusts according to the national ethnic group preterm birth rate within mums living in the most deprived areas (Index of Multiple Deprivation (IMD) 1) between April 2015 and March 2017. Figure S4b. Preterm birth (<28 weeks of gestation) rates by ethnicity across the 130 Health Trusts according to the national ethnic group preterm birth rate within mums living in the least deprived areas (Index of Multiple Deprivation (IMD) 5) between April 2015 and March 2017. Figure S5a. Preterm birth (<34 weeks of gestation) rates by ethnicity across the 130 Health Trusts according to the national ethnic group preterm birth rate within mums living in the most deprived areas (Index of Multiple Deprivation (IMD) 1) between April 2015 and March 2017. Figure S5b. Preterm birth (<28 weeks of gestation) rates by ethnicity across the 130 Health Trusts according to the national ethnic group preterm birth rate within mums living in the least deprived areas (Index of Multiple Deprivation (IMD) 5) between April 2015 and March 2017. Figure S6. Preterm birth (<34 weeks of gestation) rates by ethnicity across the 130 Health Trusts according to the overall national preterm birth rate between April 2015 and March 2017. Figure S7. Preterm birth (<28 weeks of gestation) rates by ethnicity across the 130 Health Trusts according to the overall national preterm birth rate between April 2015 and March 2017. Figure S8. Preterm birth (<34 weeks of gestation) rates by ethnicity across the 130 Health Trusts according to the national ethnic group preterm birth rate between April 2015 and March 2017. Figure S9. Prete [file 12916_2024_3493_MOESM1_ESM.zip › Additional file 1 Fig S2R3.png]

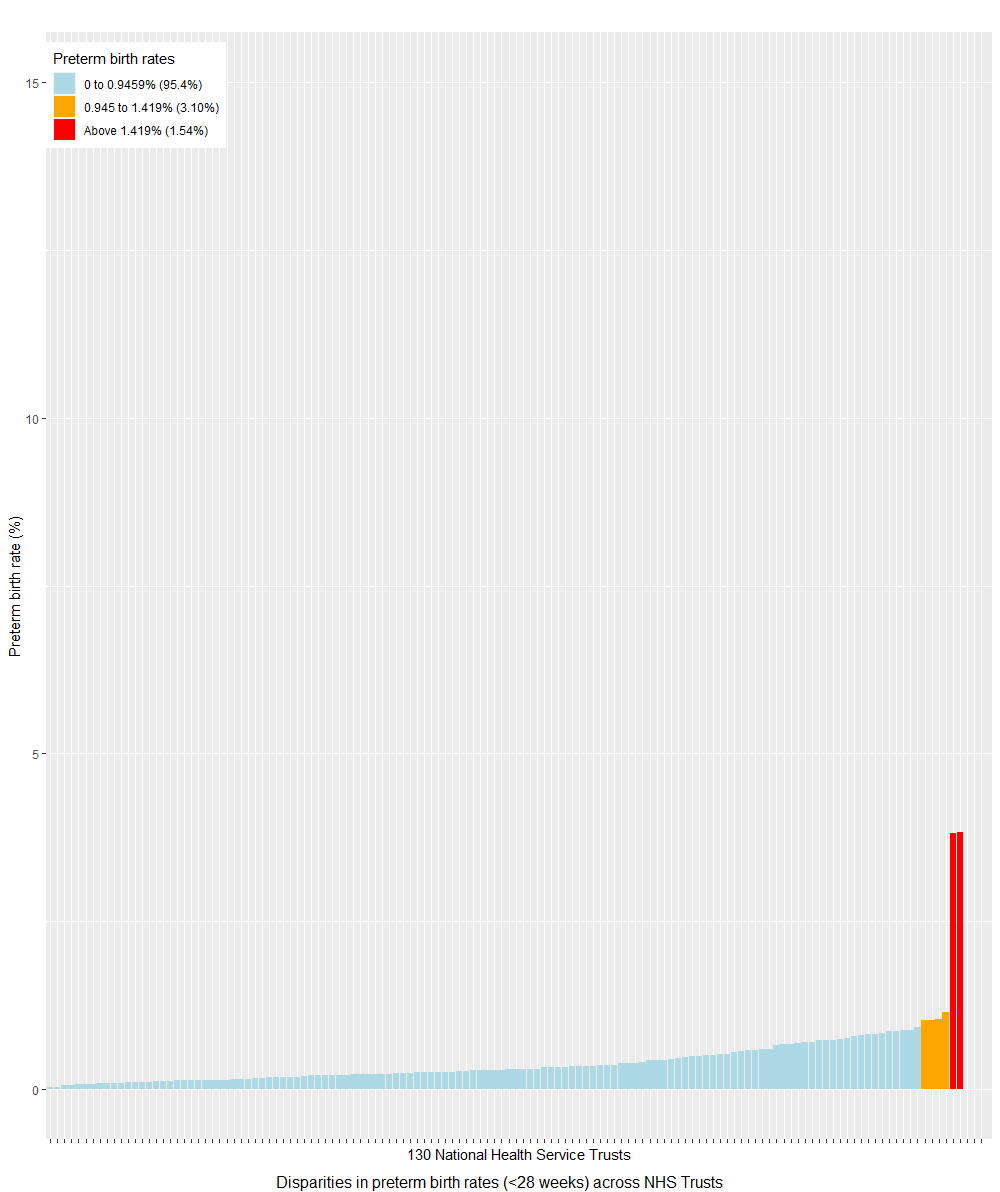

Supplement: Supplementary file 1 — Additional file 1: Figure S1. Additional file 1 Data flow diagram. Figure S2. Preterm birth (<34 weeks of gestation) rates across the 130 Health Trusts between April 2015 and March 2017. Figure S3. Preterm birth (<28 weeks of gestation) rates across the 130 Health Trusts between April 2015 and March 2017. Figure S4a. Preterm birth (<34 weeks of gestation) rates by ethnicity across the 130 Health Trusts according to the national ethnic group preterm birth rate within mums living in the most deprived areas (Index of Multiple Deprivation (IMD) 1) between April 2015 and March 2017. Figure S4b. Preterm birth (<28 weeks of gestation) rates by ethnicity across the 130 Health Trusts according to the national ethnic group preterm birth rate within mums living in the least deprived areas (Index of Multiple Deprivation (IMD) 5) between April 2015 and March 2017. Figure S5a. Preterm birth (<34 weeks of gestation) rates by ethnicity across the 130 Health Trusts according to the national ethnic group preterm birth rate within mums living in the most deprived areas (Index of Multiple Deprivation (IMD) 1) between April 2015 and March 2017. Figure S5b. Preterm birth (<28 weeks of gestation) rates by ethnicity across the 130 Health Trusts according to the national ethnic group preterm birth rate within mums living in the least deprived areas (Index of Multiple Deprivation (IMD) 5) between April 2015 and March 2017. Figure S6. Preterm birth (<34 weeks of gestation) rates by ethnicity across the 130 Health Trusts according to the overall national preterm birth rate between April 2015 and March 2017. Figure S7. Preterm birth (<28 weeks of gestation) rates by ethnicity across the 130 Health Trusts according to the overall national preterm birth rate between April 2015 and March 2017. Figure S8. Preterm birth (<34 weeks of gestation) rates by ethnicity across the 130 Health Trusts according to the national ethnic group preterm birth rate between April 2015 and March 2017. Figure S9. Prete [file 12916_2024_3493_MOESM1_ESM.zip › Additional file 1 Fig S3R3.png]

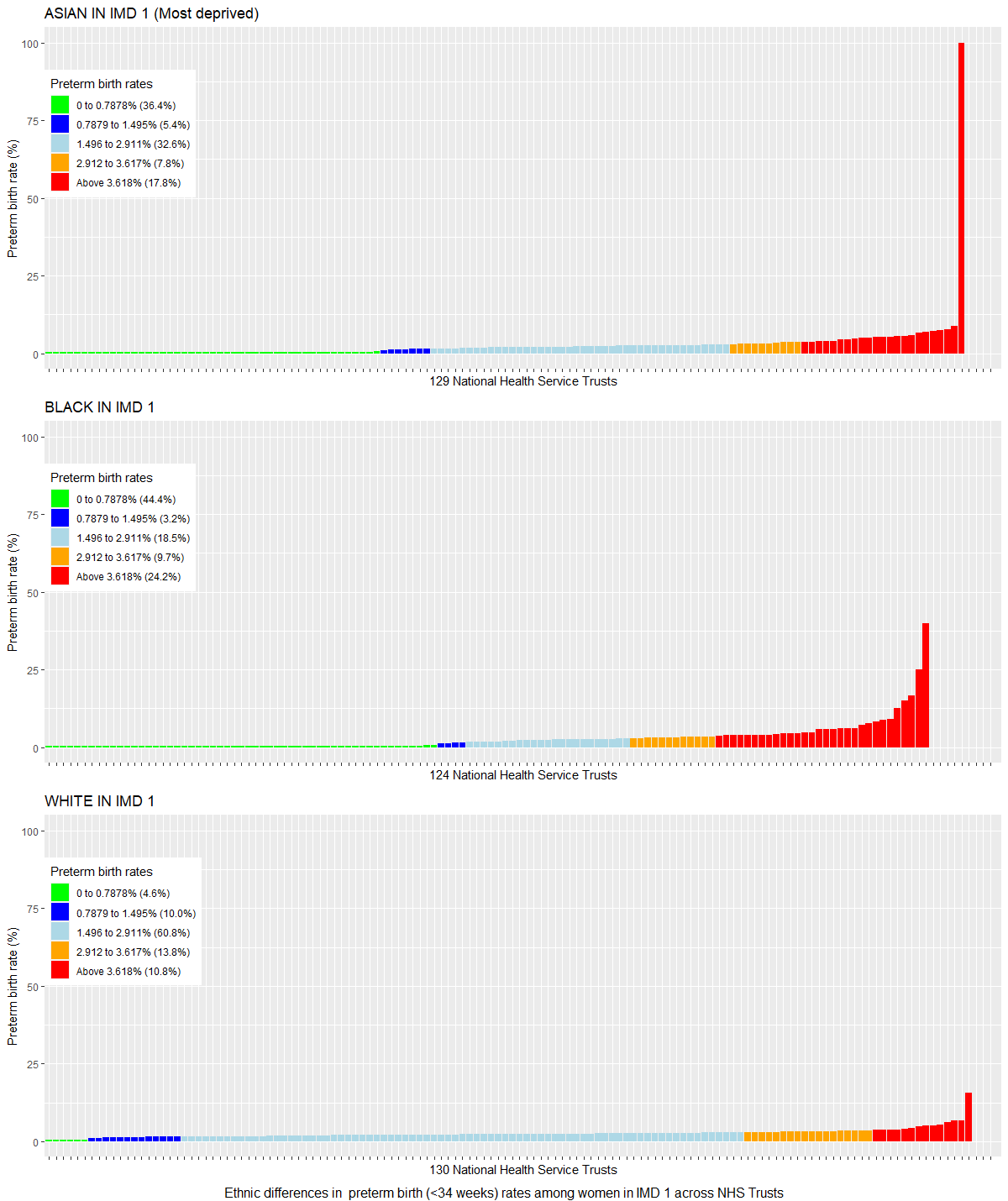

Supplement: Supplementary file 1 — Additional file 1: Figure S1. Additional file 1 Data flow diagram. Figure S2. Preterm birth (<34 weeks of gestation) rates across the 130 Health Trusts between April 2015 and March 2017. Figure S3. Preterm birth (<28 weeks of gestation) rates across the 130 Health Trusts between April 2015 and March 2017. Figure S4a. Preterm birth (<34 weeks of gestation) rates by ethnicity across the 130 Health Trusts according to the national ethnic group preterm birth rate within mums living in the most deprived areas (Index of Multiple Deprivation (IMD) 1) between April 2015 and March 2017. Figure S4b. Preterm birth (<28 weeks of gestation) rates by ethnicity across the 130 Health Trusts according to the national ethnic group preterm birth rate within mums living in the least deprived areas (Index of Multiple Deprivation (IMD) 5) between April 2015 and March 2017. Figure S5a. Preterm birth (<34 weeks of gestation) rates by ethnicity across the 130 Health Trusts according to the national ethnic group preterm birth rate within mums living in the most deprived areas (Index of Multiple Deprivation (IMD) 1) between April 2015 and March 2017. Figure S5b. Preterm birth (<28 weeks of gestation) rates by ethnicity across the 130 Health Trusts according to the national ethnic group preterm birth rate within mums living in the least deprived areas (Index of Multiple Deprivation (IMD) 5) between April 2015 and March 2017. Figure S6. Preterm birth (<34 weeks of gestation) rates by ethnicity across the 130 Health Trusts according to the overall national preterm birth rate between April 2015 and March 2017. Figure S7. Preterm birth (<28 weeks of gestation) rates by ethnicity across the 130 Health Trusts according to the overall national preterm birth rate between April 2015 and March 2017. Figure S8. Preterm birth (<34 weeks of gestation) rates by ethnicity across the 130 Health Trusts according to the national ethnic group preterm birth rate between April 2015 and March 2017. Figure S9. Prete [file 12916_2024_3493_MOESM1_ESM.zip › Additional file 1 Fig S4aR3.png]

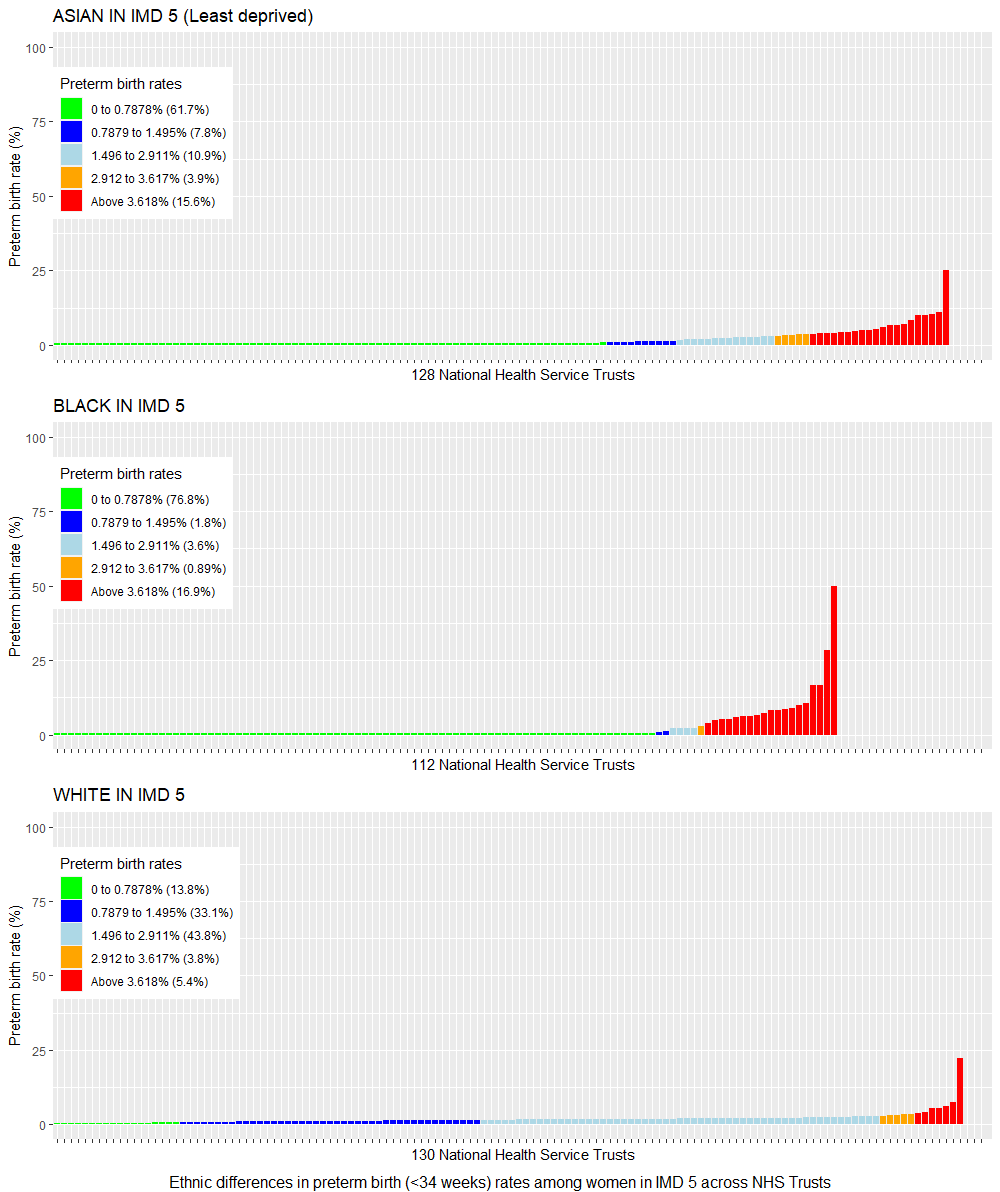

Supplement: Supplementary file 1 — Additional file 1: Figure S1. Additional file 1 Data flow diagram. Figure S2. Preterm birth (<34 weeks of gestation) rates across the 130 Health Trusts between April 2015 and March 2017. Figure S3. Preterm birth (<28 weeks of gestation) rates across the 130 Health Trusts between April 2015 and March 2017. Figure S4a. Preterm birth (<34 weeks of gestation) rates by ethnicity across the 130 Health Trusts according to the national ethnic group preterm birth rate within mums living in the most deprived areas (Index of Multiple Deprivation (IMD) 1) between April 2015 and March 2017. Figure S4b. Preterm birth (<28 weeks of gestation) rates by ethnicity across the 130 Health Trusts according to the national ethnic group preterm birth rate within mums living in the least deprived areas (Index of Multiple Deprivation (IMD) 5) between April 2015 and March 2017. Figure S5a. Preterm birth (<34 weeks of gestation) rates by ethnicity across the 130 Health Trusts according to the national ethnic group preterm birth rate within mums living in the most deprived areas (Index of Multiple Deprivation (IMD) 1) between April 2015 and March 2017. Figure S5b. Preterm birth (<28 weeks of gestation) rates by ethnicity across the 130 Health Trusts according to the national ethnic group preterm birth rate within mums living in the least deprived areas (Index of Multiple Deprivation (IMD) 5) between April 2015 and March 2017. Figure S6. Preterm birth (<34 weeks of gestation) rates by ethnicity across the 130 Health Trusts according to the overall national preterm birth rate between April 2015 and March 2017. Figure S7. Preterm birth (<28 weeks of gestation) rates by ethnicity across the 130 Health Trusts according to the overall national preterm birth rate between April 2015 and March 2017. Figure S8. Preterm birth (<34 weeks of gestation) rates by ethnicity across the 130 Health Trusts according to the national ethnic group preterm birth rate between April 2015 and March 2017. Figure S9. Prete [file 12916_2024_3493_MOESM1_ESM.zip › Additional file 1 Fig S4bR3.png]

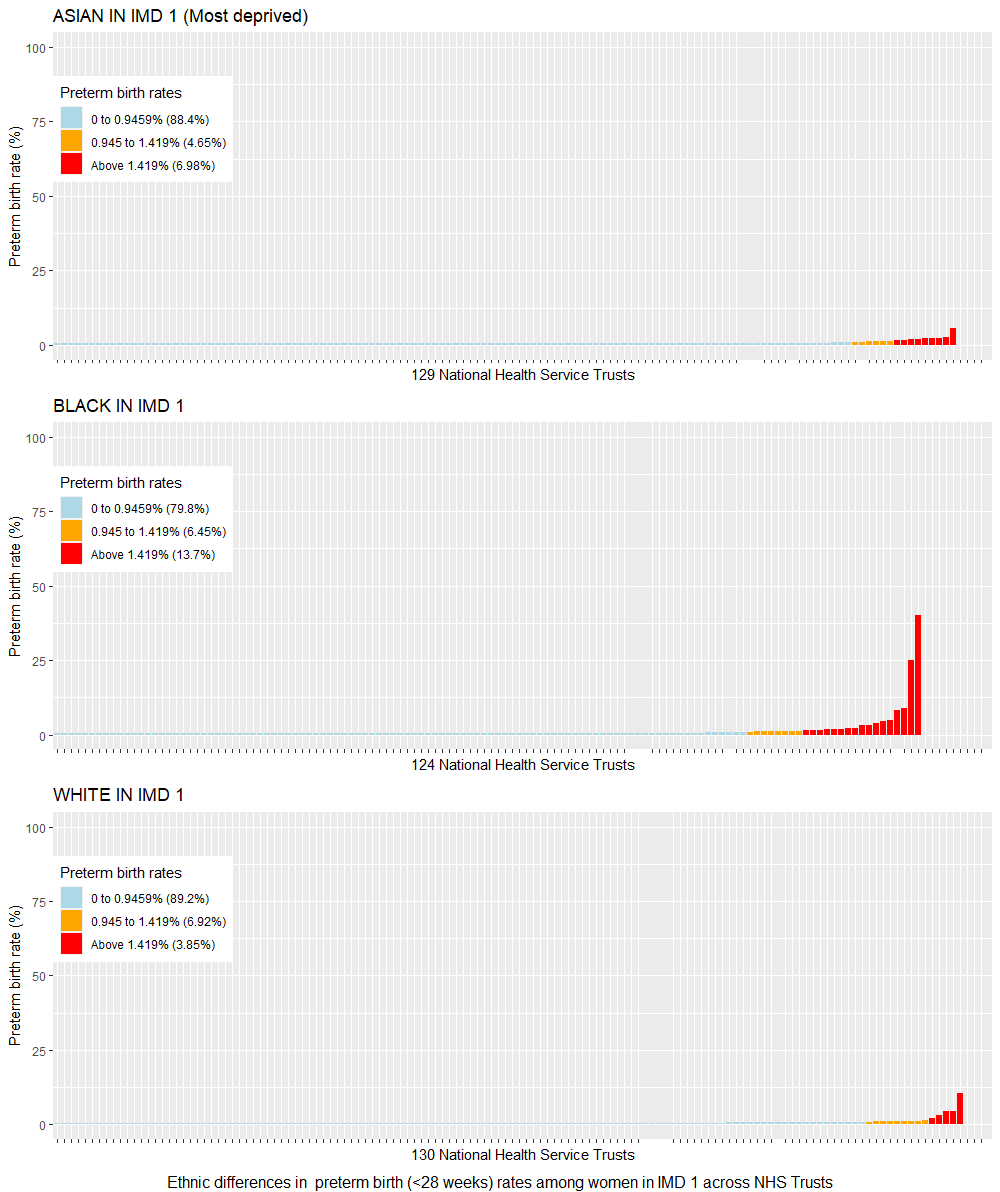

Supplement: Supplementary file 1 — Additional file 1: Figure S1. Additional file 1 Data flow diagram. Figure S2. Preterm birth (<34 weeks of gestation) rates across the 130 Health Trusts between April 2015 and March 2017. Figure S3. Preterm birth (<28 weeks of gestation) rates across the 130 Health Trusts between April 2015 and March 2017. Figure S4a. Preterm birth (<34 weeks of gestation) rates by ethnicity across the 130 Health Trusts according to the national ethnic group preterm birth rate within mums living in the most deprived areas (Index of Multiple Deprivation (IMD) 1) between April 2015 and March 2017. Figure S4b. Preterm birth (<28 weeks of gestation) rates by ethnicity across the 130 Health Trusts according to the national ethnic group preterm birth rate within mums living in the least deprived areas (Index of Multiple Deprivation (IMD) 5) between April 2015 and March 2017. Figure S5a. Preterm birth (<34 weeks of gestation) rates by ethnicity across the 130 Health Trusts according to the national ethnic group preterm birth rate within mums living in the most deprived areas (Index of Multiple Deprivation (IMD) 1) between April 2015 and March 2017. Figure S5b. Preterm birth (<28 weeks of gestation) rates by ethnicity across the 130 Health Trusts according to the national ethnic group preterm birth rate within mums living in the least deprived areas (Index of Multiple Deprivation (IMD) 5) between April 2015 and March 2017. Figure S6. Preterm birth (<34 weeks of gestation) rates by ethnicity across the 130 Health Trusts according to the overall national preterm birth rate between April 2015 and March 2017. Figure S7. Preterm birth (<28 weeks of gestation) rates by ethnicity across the 130 Health Trusts according to the overall national preterm birth rate between April 2015 and March 2017. Figure S8. Preterm birth (<34 weeks of gestation) rates by ethnicity across the 130 Health Trusts according to the national ethnic group preterm birth rate between April 2015 and March 2017. Figure S9. Prete [file 12916_2024_3493_MOESM1_ESM.zip › Additional file 1 Fig S5aR3.png]

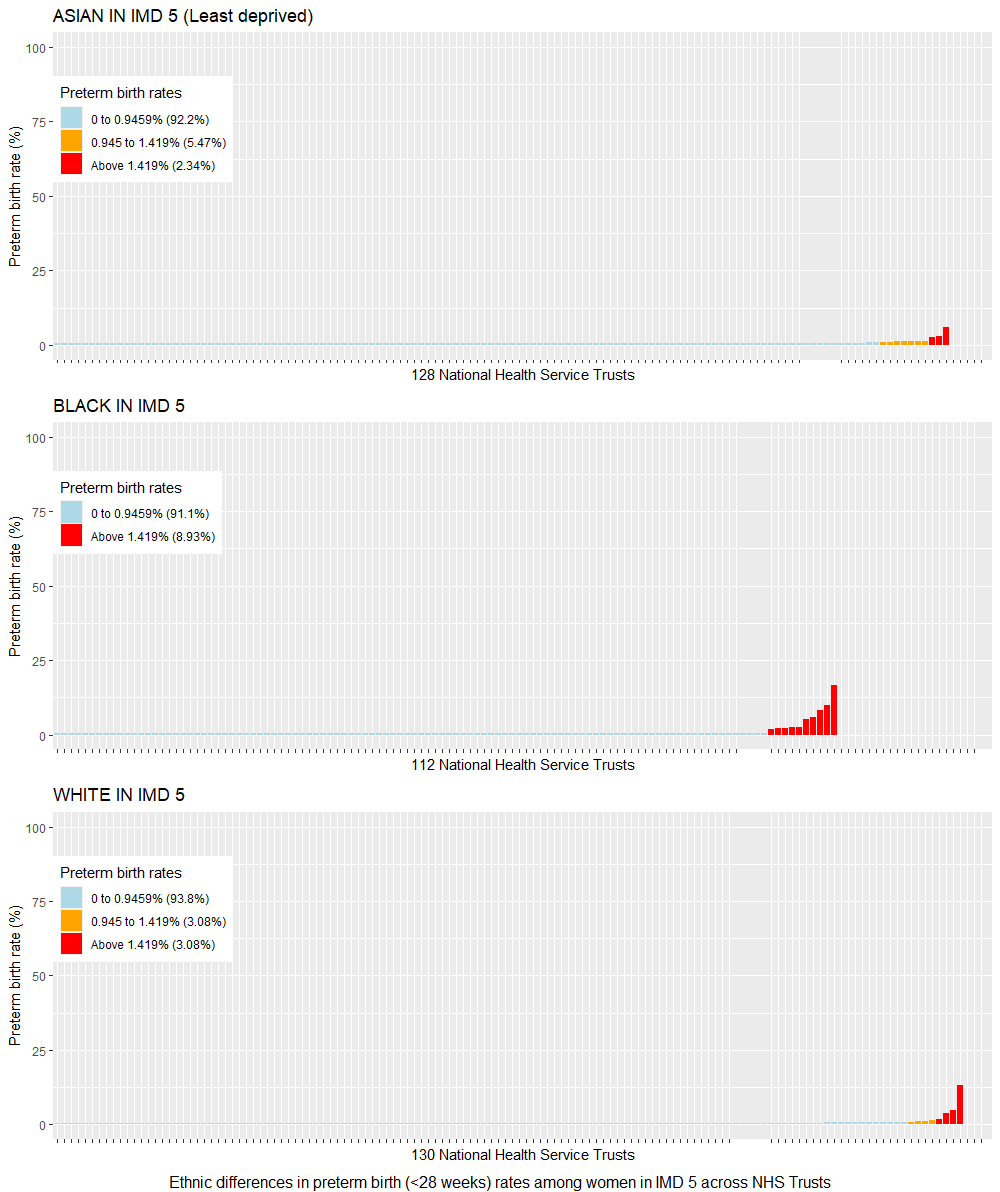

Supplement: Supplementary file 1 — Additional file 1: Figure S1. Additional file 1 Data flow diagram. Figure S2. Preterm birth (<34 weeks of gestation) rates across the 130 Health Trusts between April 2015 and March 2017. Figure S3. Preterm birth (<28 weeks of gestation) rates across the 130 Health Trusts between April 2015 and March 2017. Figure S4a. Preterm birth (<34 weeks of gestation) rates by ethnicity across the 130 Health Trusts according to the national ethnic group preterm birth rate within mums living in the most deprived areas (Index of Multiple Deprivation (IMD) 1) between April 2015 and March 2017. Figure S4b. Preterm birth (<28 weeks of gestation) rates by ethnicity across the 130 Health Trusts according to the national ethnic group preterm birth rate within mums living in the least deprived areas (Index of Multiple Deprivation (IMD) 5) between April 2015 and March 2017. Figure S5a. Preterm birth (<34 weeks of gestation) rates by ethnicity across the 130 Health Trusts according to the national ethnic group preterm birth rate within mums living in the most deprived areas (Index of Multiple Deprivation (IMD) 1) between April 2015 and March 2017. Figure S5b. Preterm birth (<28 weeks of gestation) rates by ethnicity across the 130 Health Trusts according to the national ethnic group preterm birth rate within mums living in the least deprived areas (Index of Multiple Deprivation (IMD) 5) between April 2015 and March 2017. Figure S6. Preterm birth (<34 weeks of gestation) rates by ethnicity across the 130 Health Trusts according to the overall national preterm birth rate between April 2015 and March 2017. Figure S7. Preterm birth (<28 weeks of gestation) rates by ethnicity across the 130 Health Trusts according to the overall national preterm birth rate between April 2015 and March 2017. Figure S8. Preterm birth (<34 weeks of gestation) rates by ethnicity across the 130 Health Trusts according to the national ethnic group preterm birth rate between April 2015 and March 2017. Figure S9. Prete [file 12916_2024_3493_MOESM1_ESM.zip › Additional file 1 Fig S5bR3.png]

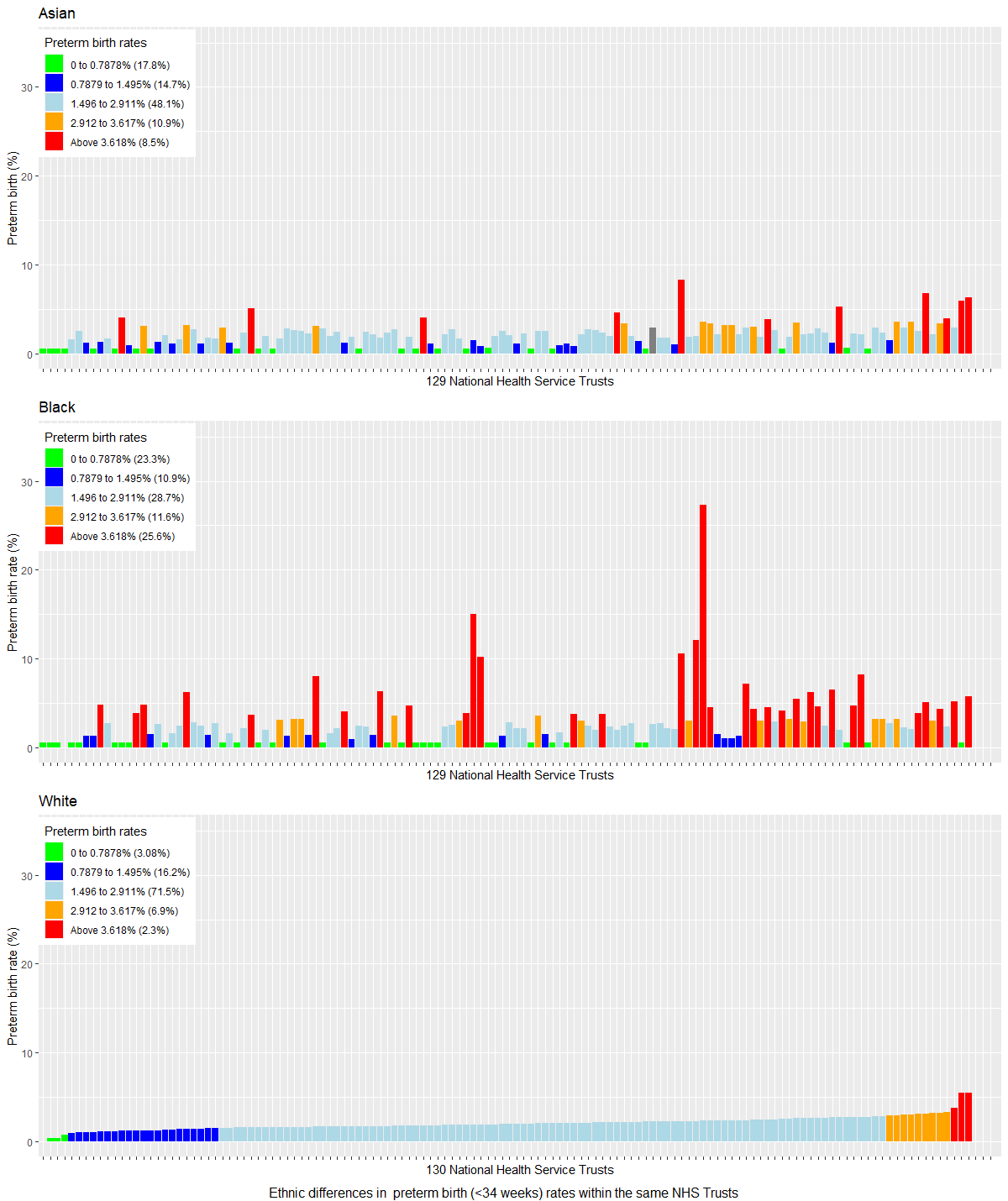

Supplement: Supplementary file 1 — Additional file 1: Figure S1. Additional file 1 Data flow diagram. Figure S2. Preterm birth (<34 weeks of gestation) rates across the 130 Health Trusts between April 2015 and March 2017. Figure S3. Preterm birth (<28 weeks of gestation) rates across the 130 Health Trusts between April 2015 and March 2017. Figure S4a. Preterm birth (<34 weeks of gestation) rates by ethnicity across the 130 Health Trusts according to the national ethnic group preterm birth rate within mums living in the most deprived areas (Index of Multiple Deprivation (IMD) 1) between April 2015 and March 2017. Figure S4b. Preterm birth (<28 weeks of gestation) rates by ethnicity across the 130 Health Trusts according to the national ethnic group preterm birth rate within mums living in the least deprived areas (Index of Multiple Deprivation (IMD) 5) between April 2015 and March 2017. Figure S5a. Preterm birth (<34 weeks of gestation) rates by ethnicity across the 130 Health Trusts according to the national ethnic group preterm birth rate within mums living in the most deprived areas (Index of Multiple Deprivation (IMD) 1) between April 2015 and March 2017. Figure S5b. Preterm birth (<28 weeks of gestation) rates by ethnicity across the 130 Health Trusts according to the national ethnic group preterm birth rate within mums living in the least deprived areas (Index of Multiple Deprivation (IMD) 5) between April 2015 and March 2017. Figure S6. Preterm birth (<34 weeks of gestation) rates by ethnicity across the 130 Health Trusts according to the overall national preterm birth rate between April 2015 and March 2017. Figure S7. Preterm birth (<28 weeks of gestation) rates by ethnicity across the 130 Health Trusts according to the overall national preterm birth rate between April 2015 and March 2017. Figure S8. Preterm birth (<34 weeks of gestation) rates by ethnicity across the 130 Health Trusts according to the national ethnic group preterm birth rate between April 2015 and March 2017. Figure S9. Prete [file 12916_2024_3493_MOESM1_ESM.zip › Additional file 1 Fig S6R3.png]

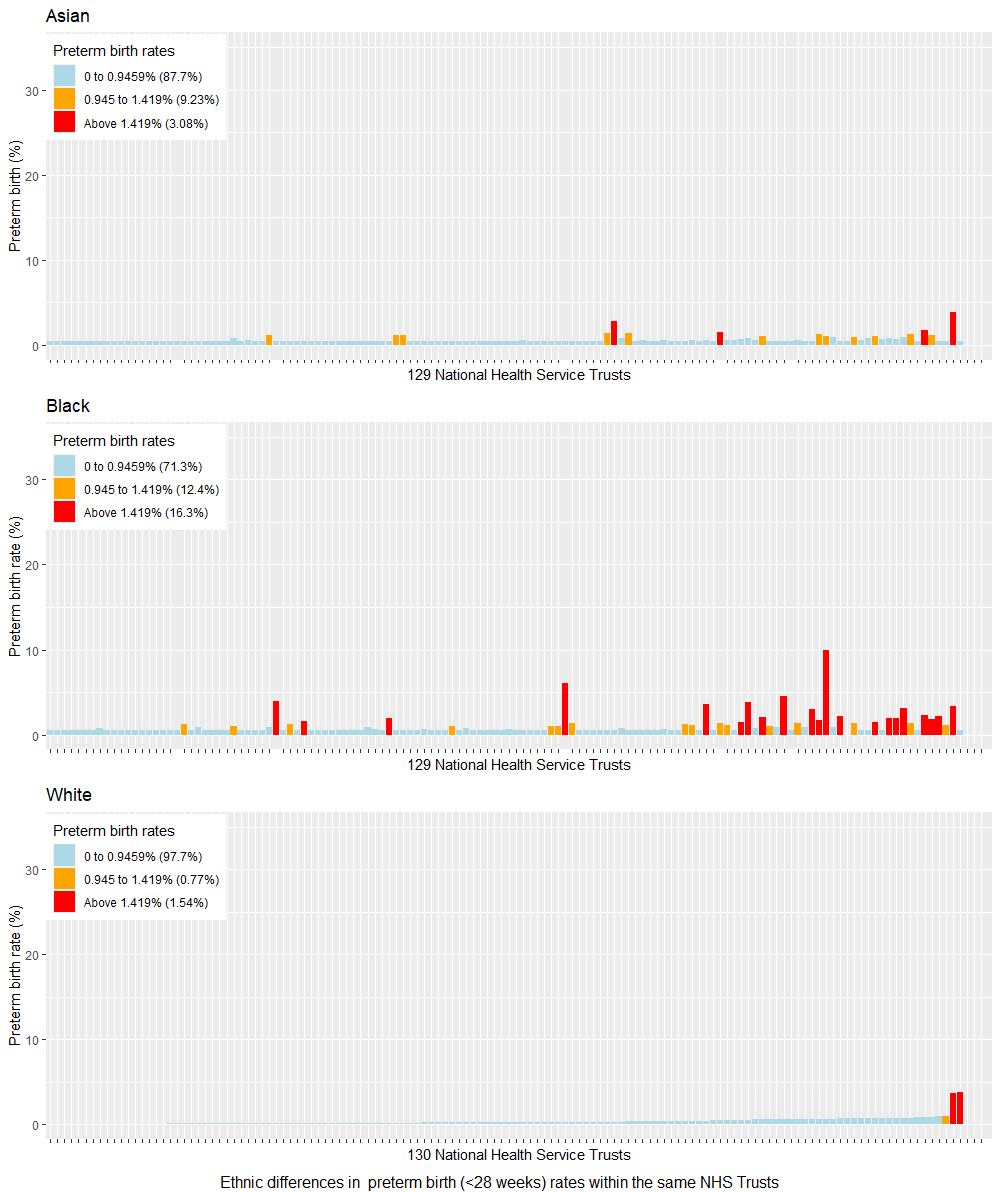

Supplement: Supplementary file 1 — Additional file 1: Figure S1. Additional file 1 Data flow diagram. Figure S2. Preterm birth (<34 weeks of gestation) rates across the 130 Health Trusts between April 2015 and March 2017. Figure S3. Preterm birth (<28 weeks of gestation) rates across the 130 Health Trusts between April 2015 and March 2017. Figure S4a. Preterm birth (<34 weeks of gestation) rates by ethnicity across the 130 Health Trusts according to the national ethnic group preterm birth rate within mums living in the most deprived areas (Index of Multiple Deprivation (IMD) 1) between April 2015 and March 2017. Figure S4b. Preterm birth (<28 weeks of gestation) rates by ethnicity across the 130 Health Trusts according to the national ethnic group preterm birth rate within mums living in the least deprived areas (Index of Multiple Deprivation (IMD) 5) between April 2015 and March 2017. Figure S5a. Preterm birth (<34 weeks of gestation) rates by ethnicity across the 130 Health Trusts according to the national ethnic group preterm birth rate within mums living in the most deprived areas (Index of Multiple Deprivation (IMD) 1) between April 2015 and March 2017. Figure S5b. Preterm birth (<28 weeks of gestation) rates by ethnicity across the 130 Health Trusts according to the national ethnic group preterm birth rate within mums living in the least deprived areas (Index of Multiple Deprivation (IMD) 5) between April 2015 and March 2017. Figure S6. Preterm birth (<34 weeks of gestation) rates by ethnicity across the 130 Health Trusts according to the overall national preterm birth rate between April 2015 and March 2017. Figure S7. Preterm birth (<28 weeks of gestation) rates by ethnicity across the 130 Health Trusts according to the overall national preterm birth rate between April 2015 and March 2017. Figure S8. Preterm birth (<34 weeks of gestation) rates by ethnicity across the 130 Health Trusts according to the national ethnic group preterm birth rate between April 2015 and March 2017. Figure S9. Prete [file 12916_2024_3493_MOESM1_ESM.zip › Additional file 1 Fig S7R3.png]

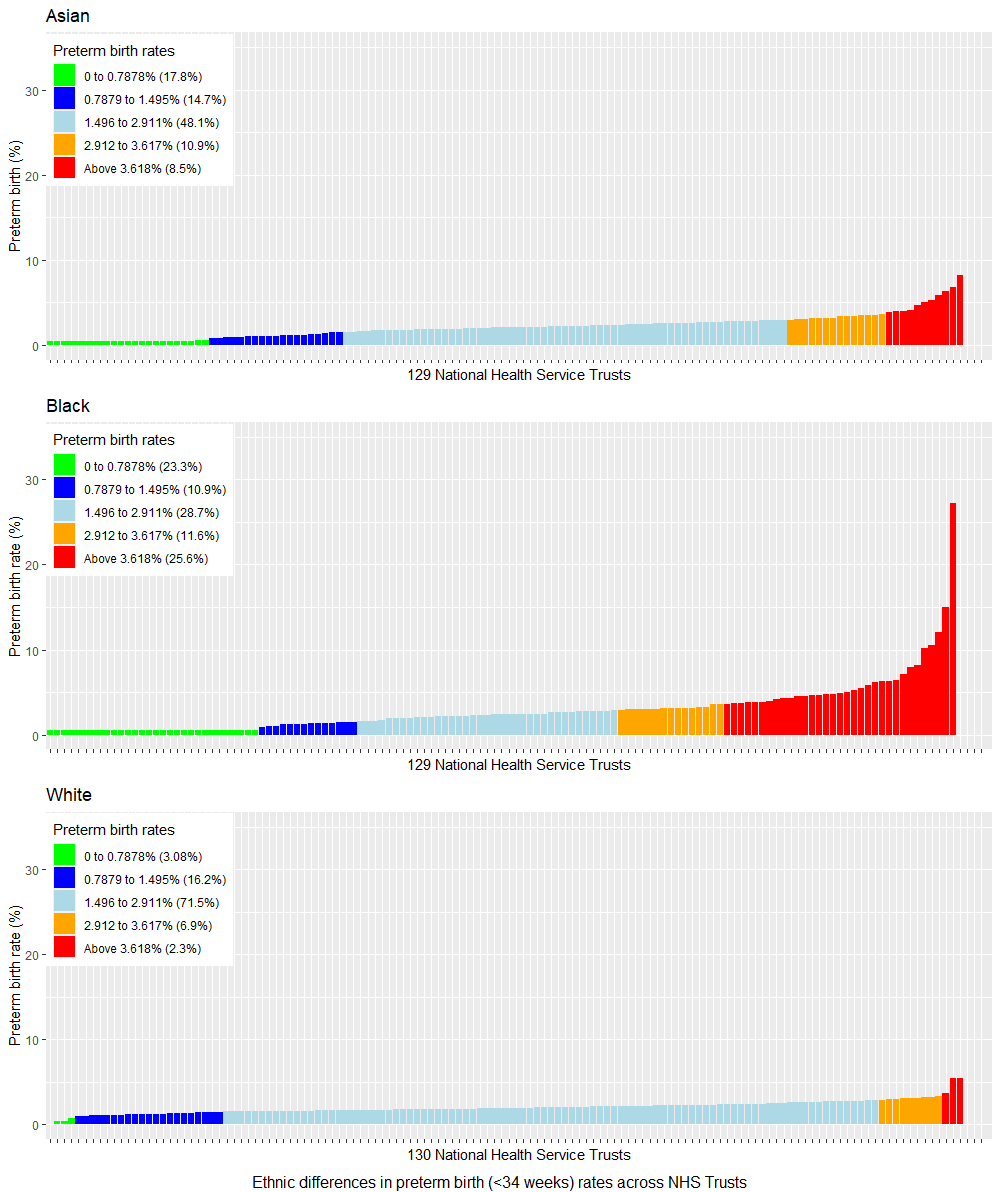

Supplement: Supplementary file 1 — Additional file 1: Figure S1. Additional file 1 Data flow diagram. Figure S2. Preterm birth (<34 weeks of gestation) rates across the 130 Health Trusts between April 2015 and March 2017. Figure S3. Preterm birth (<28 weeks of gestation) rates across the 130 Health Trusts between April 2015 and March 2017. Figure S4a. Preterm birth (<34 weeks of gestation) rates by ethnicity across the 130 Health Trusts according to the national ethnic group preterm birth rate within mums living in the most deprived areas (Index of Multiple Deprivation (IMD) 1) between April 2015 and March 2017. Figure S4b. Preterm birth (<28 weeks of gestation) rates by ethnicity across the 130 Health Trusts according to the national ethnic group preterm birth rate within mums living in the least deprived areas (Index of Multiple Deprivation (IMD) 5) between April 2015 and March 2017. Figure S5a. Preterm birth (<34 weeks of gestation) rates by ethnicity across the 130 Health Trusts according to the national ethnic group preterm birth rate within mums living in the most deprived areas (Index of Multiple Deprivation (IMD) 1) between April 2015 and March 2017. Figure S5b. Preterm birth (<28 weeks of gestation) rates by ethnicity across the 130 Health Trusts according to the national ethnic group preterm birth rate within mums living in the least deprived areas (Index of Multiple Deprivation (IMD) 5) between April 2015 and March 2017. Figure S6. Preterm birth (<34 weeks of gestation) rates by ethnicity across the 130 Health Trusts according to the overall national preterm birth rate between April 2015 and March 2017. Figure S7. Preterm birth (<28 weeks of gestation) rates by ethnicity across the 130 Health Trusts according to the overall national preterm birth rate between April 2015 and March 2017. Figure S8. Preterm birth (<34 weeks of gestation) rates by ethnicity across the 130 Health Trusts according to the national ethnic group preterm birth rate between April 2015 and March 2017. Figure S9. Prete [file 12916_2024_3493_MOESM1_ESM.zip › Additional file 1 Fig S8R3.png]

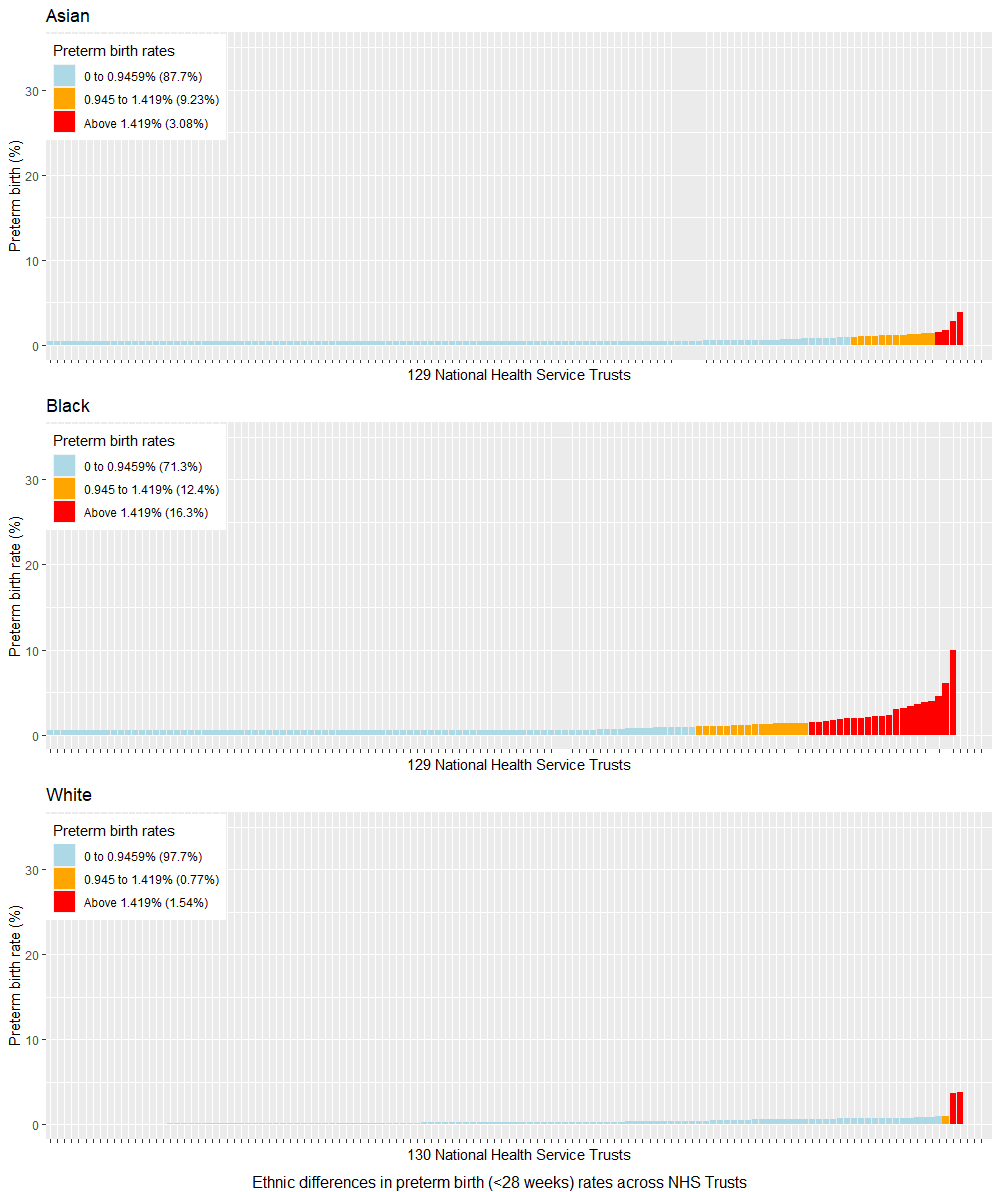

Supplement: Supplementary file 1 — Additional file 1: Figure S1. Additional file 1 Data flow diagram. Figure S2. Preterm birth (<34 weeks of gestation) rates across the 130 Health Trusts between April 2015 and March 2017. Figure S3. Preterm birth (<28 weeks of gestation) rates across the 130 Health Trusts between April 2015 and March 2017. Figure S4a. Preterm birth (<34 weeks of gestation) rates by ethnicity across the 130 Health Trusts according to the national ethnic group preterm birth rate within mums living in the most deprived areas (Index of Multiple Deprivation (IMD) 1) between April 2015 and March 2017. Figure S4b. Preterm birth (<28 weeks of gestation) rates by ethnicity across the 130 Health Trusts according to the national ethnic group preterm birth rate within mums living in the least deprived areas (Index of Multiple Deprivation (IMD) 5) between April 2015 and March 2017. Figure S5a. Preterm birth (<34 weeks of gestation) rates by ethnicity across the 130 Health Trusts according to the national ethnic group preterm birth rate within mums living in the most deprived areas (Index of Multiple Deprivation (IMD) 1) between April 2015 and March 2017. Figure S5b. Preterm birth (<28 weeks of gestation) rates by ethnicity across the 130 Health Trusts according to the national ethnic group preterm birth rate within mums living in the least deprived areas (Index of Multiple Deprivation (IMD) 5) between April 2015 and March 2017. Figure S6. Preterm birth (<34 weeks of gestation) rates by ethnicity across the 130 Health Trusts according to the overall national preterm birth rate between April 2015 and March 2017. Figure S7. Preterm birth (<28 weeks of gestation) rates by ethnicity across the 130 Health Trusts according to the overall national preterm birth rate between April 2015 and March 2017. Figure S8. Preterm birth (<34 weeks of gestation) rates by ethnicity across the 130 Health Trusts according to the national ethnic group preterm birth rate between April 2015 and March 2017. Figure S9. Prete [file 12916_2024_3493_MOESM1_ESM.zip › Additional file 1 Fig S9R3.png]
